# Vaccination against Borna Disease: Overview, Vaccine Virus Characterization and Investigation of Live and Inactivated Vaccines

**DOI:** 10.3390/v14122706

**Published:** 2022-12-02

**Authors:** Ralf Dürrwald, Jolanta Kolodziejek, Djin-Ye Oh, Sibylle Herzog, Heinrich Liebermann, Nikolaus Osterrieder, Norbert Nowotny

**Affiliations:** 1 Unit 17: Influenza and Other Viruses of the Respiratory Tract, Department of Infectious Diseases, Robert Koch Institute, Seestraße 10, 13353 Berlin, Germany; 2 Institute of Virology, University of Veterinary Medicine Vienna, 1210 Vienna, Austria; 3 Institute of Virology, Justus-Liebig-University Giessen, 35392 Giessen, Germany; 4 retd., former Institute of Microbiology and Infectious Diseases, Faculty of Veterinary Medicine, University of Leipzig, 04103 Leipzig, Germany; 5 Institut für Virologie, Freie Universität Berlin, 14163 Berlin, Germany; 6 Department of Basic Medical Sciences, College of Medicine, Mohammed Bin Rashid University of Medicine and Health Sciences, Dubai P.O. Box 505055, United Arab Emirates

**Keywords:** Borna disease, vaccine viruses, live and inactivated vaccines

## Abstract

(1) Background: Vaccination of horses and sheep against Borna disease (BD) was common in endemic areas of Germany in the 20th century but was abandoned in the early 1990s. The recent occurrence of fatal cases of human encephalitis due to Borna disease virus 1 (BoDV-1) has rekindled the interest in vaccination. (2) Methods: The full genomes of the BD live vaccine viruses “Dessau” and “Giessen” were sequenced and analyzed for the first time. All vaccination experiments followed a proof-of-concept approach. Dose-titration infection experiments were performed in rabbits, based on both cell culture- and brain-derived viruses at various doses. Inactivated vaccines against BD were produced from concentrated cell culture supernatants and investigated in rabbits and horses. The BoDV-1 live vaccine “Dessau” was administered to horses and antibody profiles were determined. (3) Results: The BD live vaccine viruses “Dessau” and “Giessen” belong to clusters 3 and 4 of BoDV-1. Whereas the “Giessen” virus does not differ substantially from field viruses, the “Dessau” virus shows striking differences in the M gene and the N-terminal part of the G gene. Rabbits infected with high doses of cell-cultured virus developed neutralizing antibodies and were protected from disease, whereas rabbits infected with low doses of cell-cultured virus, or with brain-derived virus did not. Inactivated vaccines were administered to rabbits and horses, following pre-defined vaccination schemes consisting of three vaccine doses of either adjuvanted or nonadjuvanted inactivated virus. Their immunogenicity and protective efficacy were compared to the BD live vaccine “Dessau”. Seventy per cent of horses vaccinated with the BD live vaccine “Dessau” developed neutralizing antibodies after vaccination. (4) Conclusion: Despite a complex evasion of immunological responses by bornaviruses, some vaccination approaches can protect against clinical disease. For optimal effectiveness, vaccines should be administered at high doses, following vaccination schemes consisting of three vaccine doses as basic immunization. Further investigations are necessary in order to investigate and improve protection against infection and to avoid side effects.

## 1. Introduction

Borna disease (BD) is caused by Borna disease virus 1 (BoDV-1) or 2 (BoDV-2) which mainly affect the central nervous system of mammals [1]. Other bornaviruses have been identified in birds, snakes and fish [2]. Bornaviruses are nonsegmented, negative-sense single-stranded RNA viruses of about 8.9 kb in size, classified in the family *Bornaviridae* of the order *Mononegavirales* [3]. The existence of endogenous virus sequences in the genomes of many vertebrates indicates multiple infection events with related viruses during evolution [4,5]. BoDV-1 and BoDV-2 are noncytopathic and capable of establishing persistence, using diverse mechanisms [1], which include: (i) replication in the nucleus, enabling the binding of viral replication complexes onto the host cell chromatin [6]; (ii) genome trimming in order to generate RNAs that are unlikely to trigger innate immune responses in infected cells [7,8]; (iii) suppression of apoptosis in infected cells mediated by the accessory protein (X), which lead to persistent noncytolytic infection [9,10], and (iv) low-level expression of the glycoprotein G [11].

The Bornavirus genome is organized into three transcription units and contains at least six ORFs in the order 3′-N-X/P-M-G-L-5′ [2]. The transcription unit 3 of orthobornaviruses has at least two introns [12,13]. The expression of BoDV-1 M (matrix), G, and L (polymerase) is performed through differential splicing of the two introns and leaky ribosomal scanning, using the cellular splicing apparatus [13,14,15,16]. BoDV-1 G is encoded by two mRNAs, which contain up to three exons encoding M, G, and L [14,17,18,19,20]. Translation of G mainly occurs using mRNA isoforms, lacking intron I but retaining intron II [14,16,20]. G is post-translationally modified by *N*-glycosylation and undergoes proteolytic cleavage by cellular subtilisin-like proteases, like furin, resulting in the N-terminal and C-terminal cleavage products GP-N and GP-C, respectively [21].

BD occurs in horses and sheep in several federal states of Germany, in particular Bavaria, parts of Saxony, Saxony-Anhalt, Thuringia and Lower Saxony [22]. Over the last decades the disease was also detected in Austria, Switzerland and The Principality of Liechtenstein, as well as in other German federal states such as Brandenburg [23,24].

BD was first seen in the Swabian Alps in the mid19th century but may have existed before [25]. By end of the 19th century, disease outbreaks in horses and sheep in Saxony led to the first scientific investigations, which revealed a pathology of nonpurulent encephalitis [26]. The frequent occurrence of sporadic cases in western Saxony, with a concentration around the city of Borna, led farmers and veterinary practitioners to name the disease as Borna disease. Although the disease may have been more prevalent in other parts of Germany, such as Bavaria, southern parts of Saxony-Anhalt and Thuringia, this is the designation that prevailed, presumably because it was short and sonorous [22].

In the 1920s the viral etiology was established by Wilhelm Zwick and his co-workers in Giessen, based on infection experiments in rabbits [27,28]. This sparked the development of the first vaccines [29,30] (see Supplementary Material S1 [28,29,31,32,33,34,35,36,37,38,39,40,41,42,43,44,45,46,47,48,49,50,51,52,53,54,55,56,57,58,59,60,61,62]). When trials of inactivated vaccines failed [61], a live vaccine approach was pursued [30]. Given the noncytolytic nature of the virus, cell culture propagation was not an option at the time, instead, brain material of infected animals was used for live vaccination. The brains of infected horses were widely used for vaccination in Southern Germany in the 1930s and 1940s, but this practice was abolished following the unintended transmission of extraneous agents including infectious anemia virus by this procedure [35]. Therefore, mostly brains of experimentally infected rabbits were used for vaccinations. Due to the substantial economic burden of BD in horses, this vaccination approach became widespread in endemic areas of Germany in the late 1920s and 1930s, especially in the province Saxony of Prussia, in Bavaria and in adjacent Baden-Wurttemberg. After World War II and the division of Germany, the practice of vaccination against BoDV-1 was continued. In West Germany, vaccines were based on Zwick’s vaccine virus, which had been established in the 1920s/1930s by passaging in rabbits. They were regularly administered until the 1980s, when West Germany abolished BoDV-1 live vaccines given regulatory concerns for adverse effects due to potentially insufficient attenuation [32]. In East Germany, a similar vaccination approach was pursued in the city of Dessau, where, during the late 1940s and early 1950s, different viruses from diseased sheep and horses were passaged in rabbits by experimental infection. Brain material from these rabbits was used to produce the Borna live vaccine “Dessau”, which was applied for subcutaneous vaccination of horses and sheep in East Germany until 1992. This vaccine was only authorized in the German Democratic Republic and its distribution was no longer sustained after the German reunification (sale of the last batch was restricted to 1992). Given the decrease in horse population since the 1960s, BD had become considerably less important, thus, vaccine development was not pursued further after 1992 (see Supplementary Material S2 [1,31,46,58,63,64,65,66,67,68,69,70,71]).

Meanwhile several vector-based vaccination approaches were conducted successfully at scientific level [39,41,52,56,72]. There are two major ways to be considered in vaccination: (i) elimination of BoDV-1 from the body infected before entry into cells of the central-nervous system [73] and (ii) elimination of BoDV-1 from infected cells [41]. It has been shown that neutralizing antibodies (mainly directed against G) can prevent nerve cell entry when provided before infection [73]. Antiviral CD8+ T cells can act against infected cells. This antiviral activity of primed T-cells is critical because it can enhance disease. If the primary stimulation of T-cells is weak, immunization can contribute to an exacerbation of BD during the course of vaccination [42]. Strong stimulation by several vaccine doses is a precondition for successful immunization against BD [41].

In the last few decades, a wealth of new findings on Borna disease has emerged, it was found in a BD-endemic area in Switzerland that white-toothed bicoloured shrews harbor the virus without developing disease and thus may play a role in virus transmission [74]. Other bornaviruses were discovered in birds, among them the etiologic agent of proventricular dilation disease [75]. The discovery of a new mammalian bornavirus, Variegated squirrel bornavirus 1, was associated with fatal cases of encephalitis in humans, which could be traced back to persistent infections in squirrels [76,77]. Finally, it was recently demonstrated that sporadic cases of fatal encephalitis in humans were caused by BoDV-1, the agent of BD in horses [78,79,80,81,82].

The possibility of zoonotic transmission of BoDV-1 was first discussed in the 1920s [83,84] and again after the detection of antibodies in humans in the 1980s [85] and 1990s [86], subsequently, interest in this discussion faded, potentially due to an unsuccessful approach to link BoDV-1 infection with psychiatric disease [87]. BD has meanwhile been diagnosed in several human encephalitis patients in most of the endemic regions in Germany [88]. The transmission route to humans and animals is still unknown, which limits the effectiveness of infection prevention [89]. Thus, effective vaccination approaches may play a relevant role in the development of future preventive health strategies.

The goals of this study were: (i) to provide a detailed history of vaccines and vaccination strategies against BoDV-1, based on a few (mainly in German language) published and many unpublished studies and observations, and (ii) to establish the complete genome sequences of two live vaccine strains and their placement within BoDV-1 phylogeny.

## 2. Materials and Methods

### 2.1. Viruses

The following viruses were used for our investigations: (i) for infectious dose titration experiments in rabbits and the production of experimental batches of inactivated vaccines used in rabbits, the Berlin variant of BoDV-1 Strain V, originally isolated by Zwick et al., in Giessen [71]; this virus was brought to Berlin in 1978 and sequenced in the 1990s [31]; (ii) for sequencing, the Giessen variant of BoDV-1 strain V, which had been used as vaccine virus in the Western part of Germany Op No. 022059 from 1948 (this paper), and (iii) for sequencing, vaccine production and horse vaccination, the vaccine virus “Dessau” from batch 198 11 90 from 1990 [46,58].

### 2.2. Vaccines

#### 2.2.1. Authorized Live Virus Vaccine

The vaccine Borna-Lebendimpfstoff “Dessau” was produced at VEB Impfstoffwerk Dessau-Tornau (later GmbH) in Tornau near Rodleben (district of Dessau, currently Dessau-Roßlau), Germany. The BoDV-1 seed lot material (established from a diseased horse, exact origin not documented) was diluted 1:30 with NaCl solution. A half mL of this solution was injected intracerebrally into rabbits of 1.5–2.0 kg body weight. Before development of signs of disease the rabbits were euthanized and brains were taken. The sterile brains (60–70 brains) were diluted 1:15 with NaCl solution. 0.1 mg Streptomycin and 150 IU Penicillin were added to this homogenate. Afterwards the homogenate was filled in glass bottles and lyophilized. Titration of the homogenate in rabbits resulted in 1 × 10^3^ Rabbit Infectious Doses 50 (RID_50_) per dose. Freedom from bacteria was proven on agar plates and bouillons. One package of the vaccine contained 5 bottles of lyophilized rabbit brain material. Added in the package was a bottle with 50 mL liquid for suspension. Using the diluent a 10 mL suspension had to be created from each bottle shortly before vaccination. The dose for subcutaneous vaccination of horses was 10 mL, for sheep 0.4 mL. The vaccine was authorized for use in horses and sheep on the territory of the German Democratic Republic. Shortly after the German reunification the authorization of the vaccine ceased on 30 June 1992 (when shelf life of the last batch produced ended).

Two batches of the BoDV-1 live vaccine “Dessau” were titrated for infectivity in Young Rabbit Brain (YRB) cells. Batch 195 05 90 contained 1 × 10^3^ focus forming units per milliliter (FFU/mL), batch 199 66 91 (the last batch of this vaccine, produced in June 1991) contained 5 × 10^3^ FFU/mL after generating a suspension of 5 mL from the lyophilized material. Thus, the dose for horses ranged between 3.7–4.4 lg FFU (5 × 10^3^–2.5 × 10^4^ FFU of BoDV-1); for sheep it ranged between 2.3–3.0 lg FFU (2 × 10^2^ FFU–1 × 10^3^ FFU). Two batches (186 03 89 and 194 03 90) were used for vaccination of horses in the spring of 1992 but were not available for titration in YRB cells.

#### 2.2.2. Experimental Batches of Vaccines

##### Vaccines Based on Strain V

BoDV-1 strain V was propagated in persistently infected OL cells (OL/TL cells) in cell culture flasks (Greiner Bio-One, Kremsmünster, Austria, cell surface 175 cm^2^) using Dulbecco’s Medium Eagle’s Modification (DMEM) as medium + 5% FBS at 37 °C. After 24 h the medium was changed and DMEM with 5% FBS supplemented with 10 mM n-butyrate (Thermo Scientific^TM^, Waltham, MA, USA, 13458488, 1.1 g/L DMEM) was added. After 48 h the medium was changed to DMEM containing 300 mM NaCl (17.5 g/L). Two hours later, the supernatant was collected and aliquots were ultracentrifuged at 100,000× *g* for 120 min. The pellets were resuspended in 1 mL PBS (pH 7.2), pooled, and further diluted with PBS. The virus content was adjusted to 10^7.1^ FFU/mL. The virus was inactivated (i) by ethylenimine or (ii) by incubation at room temperature over 6 weeks. Inactivation was confirmed by inactivation kinetics and titration in cell culture (OL cells). From the ethylenimine-inactivated suspension two different vaccines were produced by mixing the supernatant with 20% Montanide^TM^ ISA25 (Seppic GmbH, Köln, Germany) (ISA25) or 20% PBS in order to generate one adjuvanted and one not adjuvanted vaccine with same content of antigen (10^7^ FFU/1 mL). The experimental vaccines were used for vaccination and challenge infection experiments in rabbits (dose 1 mL).

##### Vaccines Based on Strain “Dessau”

After propagation of BoDV-1 virus “Dessau” in Newborn Rabbit Brain (NRB, permanent cell line established from the brains of newborn rabbits at Impfstoffwerk Dessau-Tornau, Dessau-Roßlau, Germany) cells (NRB/BDV), OL cells (OL/BDV cells), RK13 cells (RK13/BDV), MDBK cells (MDBK/BDV) the latter were chosen for cultivation of this virus in roller bottles (Corning, NY, USA, 850 cm^2^) using DMEM as medium. After 24 h the medium was changed to DMEM with 5% FBS supplemented with 10 mM n-butyrate. After 48 h, the medium was changed. to DMEM containing 300 mM NaCl. Two hours later the supernatant was taken and the virus content was determined. Proportions of the virus harvest were ultracentrifuged at 100,000× *g* for 120 min. The pellets were resuspended in 1 mL PBS (pH 7.2), pooled and further diluted with PBS. The virus content was adjusted to 10^7.1^ FFU/mL. From the suspension two different vaccines were produced by mixing the supernatant with either 20% Montanide^TM^ ISA25 (Seppic GmbH, Köln, Germany) (ISA25) or 20% PBS in order to generate one adjuvanted and one non-adjuvanted vaccine with same content of antigen (10^7^ FFU/1 mL). The experimental vaccines were used for vaccination experiments in horses (dose 1 mL) for determination of antibody levels.

##### Vaccines for Dose Titration

For dose titration of vaccines, the BoDV-1 live vaccine “Dessau” was diluted in tenfold steps (log_10_ = lg). Furthermore, the suspension produced in MDBK/BDV cells (see Vaccines based on Strain “Dessau”) was also diluted in 10-fold dilution steps and then inactivated by ethylenimine. Vaccine formulations were used with or without adjuvant in the inactivated vaccine, which were used for comparative analyses of antibody responses in rabbits.

##### Inactivation of Viruses and Inactivation Kinetics

The inactivation of BoDV-1 was carried out by adding a 0.8% solution of binary ethyleneimine (Ferak GmbH, Berlin, Germany) to a final concentration of 0.05%. Ethylemimine is an aziridine substance and does not react with proteins. Binary ethylenimine is prepared from two substances, 2-bromoethylamine Hbr and NaOH. The virus inactivation was carried out by stirring the suspension at temperatures between 18 and 25 °C for a minimum of 23 and a maximum of 48 h, at which time ethylenimine was hydrolized and, thus deactivated by addition of a 50% sodium thiosulphate solution to an end concentration of 1%. The deactivation was done by stirring at 18 °C to 25 °C for 4–8 h. Adjustment of the pH value to 7.00 ± 0.5 by addition of KH_2_PO_4_ was followed. Deactivation kinetics reflect that deactivation is a rapid process and after 3 h of deactivation enough unbound Na-thiosulphate is available.

The process of inactivation was investigated by means of inactivation kinetics. Here, the reaction was stopped with sodium thiosulphate at certain points in time to evaluate at which time point inactivation was complete (0, 1, 2, 4, 6, 8, 10, 12, 15, 23, 32, 48 h). This was done with concentrated materials with highest virus content at start. The virus titration was carried out on OL cells.

The inactivation of viruses was ascertained on the final material by passaging three times on OL-cells and staining with specific antibodies.

Furthermore, inactivation was investigated on the material containing the same amount of virus at room temperature (23 °C). For this, virus suspensions were portioned and stored at 23 °C. Each week the virus was titrated on OL cells.

### 2.3. Virus Titration

Virus titrations were done on Young Rabbit Brain (YRB) cells or Oligodendroglia (OL) cells. The second passage of YRB cells or different passages of OL cells were seeded into cavities of microtitre plates (10^4^ cells each cavity). The microtitre plates were incubated at 37 °C and 5% CO_2_ for 24 h. The material under investigation (brain suspension, cell culture supernatant, vaccine solution) underwent serial tenfold (log_10_ = lg) dilution. The dilutions were transferred to the micotitre plates with the cell monolayer. The microtitre plates were incubated at 37 °C and 5% CO_2_ over 5 d. The cells were fixed with 3% formaldehyde solution and an ELISA was performed as described elsewhere [90]. Triton X-100 solution (1% in PBS) was added into each cavity and incubated at room temperature for 30 min before cells were washed with washing solution and the monoclonal antibody Kfu2 (anti-P, 25 and 60 kD) was added [66]. After incubation at 37 °C and 5% CO_2_ for 60 min cells were washed and anti-mouse IgG peroxidase conjugate (Nordic) was added. The microtire plates were again incubated at 37 °C for 60 min. After three washing steps substrate solution (3-amino-9-ethyl-carbazol, Sigma, dimethylsulfoxide, acetate buffer, 3% H_2_O_2_) was added and incubated at room temperature over 30 min. Then, the reaction was stopped with water. The foci were counted under a light microscope.

### 2.4. Serological Investigations

For serological investigations an indirect immunofluorescent assay (IF) and a neutralization assay (NT) were employed as described before [66,86].

For IF sera were inactivated at 56 °C for 30 min. Dilutions were prepared starting at a 1:10 dilution in PBS pH 7.2 and 1% FBS containing monoclonal antibody Kfu2 (anti BoDV-1 P, 25 and 60 KD). YRB cells were infected with BoDV-1 virus V (pool from infected rat brains). In some experiments persistently infected OL cells were used (OL/TL or OL/BDV). After incubation at 37 °C and 5% CO_2_ for 5 days cells were fixed with icecold acetone or 3% formaldehyde solution. After three washing steps of the fixed cells with PBS, the serum dilutions were added and incubated at 37 °C for 45 min. Cells were then washed with PBS, and FITC-conjugated anti-horse or anti-rabbit IgG as well TRITC-labelled F(ab′)_2_ anti-mouse IgG (Dianova, Hamburg, Germany) were added and plates incubated at 37 °C for 45 min. Cells were finally analyzed using a fluorescence microscope. Sera showing the same pattern of nuclear BoDV-1 staining (FITC, green) as the monoclonal antibody (TRITC, red) were regarded positive.

For NT sera were inactivated at 56 °C for 30 min. Serum dilutions starting 1:2 were done DMEM in microtitre plates. Then, the same volume of a virus suspension containing 30–50 FFU BoDV-1 strain V in DMEM was added. The mixture was incubated at 37 °C for 60 min and YRB or OL cells were added (10^4^ cells into each cavity). The cells were incubated at 37 °C and 5% CO_2_ atmosphere for 5 days and ultimately fixed with 3% formaldehyde solution. Immunostaining was performed ([90], see Section 2.3). The 50% focus reduction was calculated in comparison to mean values in FFU of the control cells which were carried along each assay. The neutralizing dose 50 (ND_50_) corresponds to the dilution at which a 50% reduction of infected cells (stained foci) was achieved.

### 2.5. Animal Experiments and Vaccinations

Animal experiments in rabbits and rats were done at the Institut für Virologie of Freie Universität Berlin in 1994–1996 while horses and other rabbits were maintained and vaccinated at the Impfstoffwerk Dessau-Tornau in the 1990s. The studies were conducted in compliance with all the relevant local, state, and federal regulations. They were notified to and approved by the authorities responsible at the time, i.e., the Senatsverwaltung für Gesundheit Berlin (references A/0170/00 and G0152/96), the Staatliches Veterinärmedizinisches Prüfungsinstitut Berlin, the Regierungspräsidium Dessau, the Landesverwaltungsamt Sachsen-Anhalt (#1860389, #1950590, #1940390, #1981190, AZ). An overview of all animal trials is provided in Supplementary Material S3.

#### 2.5.1. Study of Vaccine Virus Virulence in Rats

Trial I. The newborn litters (7 and 8 animals) of two Wistar rats were infected intranasally with 10^3^ FFU of BoDV-1: One litter was inoculated with the live vaccine virus strain “Dessau”, obtained from the suspended vaccine solution; the other litter (control) was inoculated with BoDV-1 strain V/B, pooled after passage in rats. Both litters, the one infected with vaccine virus and the one infected with the rat-passaged virus V/B were co-housed with their mothers and observed over a period of one to two months. They were then sacrificed and investigated for the presence of virus by RT-PCR.

#### 2.5.2. Titration of BoDV-1 in Rabbits

Trial IIA. Twelve week-old New Zealand Whites (25 rabbits in total) were infected intracerebrally with different doses of cell-culture virus or brain-derived BoDV-1. Persistently BoDV-1 strain V-infected OL/TL cells were trypsinised, centrifuged, ultrasonicated and diluted in sterile PBS (pH 7.2). Suspensions of 0.4 mL containing 10^5^, 10^4^, 10^3^, 10^2^, and 10^1^ focus forming units (FFU) BoDV-1 were injected intracerebrally (i.c.) into 5 rabbits in each group. One control animal was used as an uninfected sentinel in each group.

Trial IIB. Additionally, brain material was used for infection (25 rabbits). The brain material was derived from experimentally and persistently infected rats or experimentally infected rats. Among 67 brains those with high virus titres were selected, and a pool was generated from 15 brains. The same pool of virus V was used in all experiments. Brains were ultrasonicated and suspensions were prepared as described above for cell-culture-derived virus. The skin of the rabbits was cut along the *Crista saginata* (0.5 cm) at half the distance between its caudal end and the eyes. After trepanning the inoculum was injected into the brain.

Trial III. Further experiments were done using intact and not ultrasonicated OL/TL cells (one rabbit infected with 10^5^ FFU BoDV-1), CL6/TL cells (one rabbit infected with 10^5^ FFU BoDV-1 and one rabbit infected with 10^1^ FFU BoDV-1), and YRB cells (one rabbit infected with 10^4^ FFU BoDV-1).

Body weights and symptoms were measured daily after infection. Antibodies (NT and IF) were determined every half a week after infection. Animals that developed the disease were euthanized as soon as the humane endpoint was reached when organ samples were taken and investigated for viral RNA by RT-PCR. After the study had been completed one rabbit of each group was euthanized and investigated for the presence of viral signals by RT-PCR.

Trial IV. Five rabbits were infected i.c. with 10^5^ FFU BoDV-1 (OL/TL) and thereafter euthanized in weekly intervals (1–5 dpi) for the investigation of the presence of the virus in brain regions and other organs.

#### 2.5.3. Dose Titration of Vaccines in Rabbits

Trial V. Fifty twelve weeks-old New Zealand Whites were used for titration of the vaccination dose. Of the inactivated vaccine batches which contained 10^7^ FFU, 10^5^ FFU, 10^3^ FFU, 10^1^ FFU of BoDV-1 “Dessau” before inactivation were used. An adjuvanted (ISA25) and a nonadjuvanted vaccine were compared. The titration of the BoDV-1 live vaccine was restricted to 10^3^ and 10^1^ FFU because the vaccine did not contain more than 10^3^ FFU BoDV-1 “Dessau”. Each group included 5 rabbits. The rabbits were vaccinated subcutaneously at day 0 and revaccinated with the same dose three weeks after the first administration of the vaccine and then a third time with the same dose 16 weeks after the second administration of the vaccine. Blood samples were taken 10 days after each administration of the vaccine and tested for antibodies (NT and IF).

#### 2.5.4. Proof of Efficacy of the BD Live Vaccine “Dessau” in Rabbits

Trial VI. Five twelve weeks-old New Zealand Whites and five adult rabbits of several breeds were vaccinated subcutaneously (s.c.) with the BoDV-1 live vaccine “Dessau” batch 195 05 90 (the horse dose of 3.7 FFU resuspended in 2 mL was applied). Two additional rabbits were not vaccinated. Four weeks after immunization the ten rabbits as well as two unvaccinated control rabbits were infected intracerebrally with 10^2^ FFU BoDV-1 of brain-derived BoDV-1 (virus V—twelve weeks old group and virus “Dessau”—adult group). Clinical symptoms and body weights were recorded. Blood samples were taken before vaccination and weekly until 5 weeks after vaccination.

#### 2.5.5. Investigation of Experimental Batches of Inactivated Vaccines in Rabbits

Trial VII. Five twelve weeks-old New Zealand Whites were vaccinated s.c. twice within three weeks and again 16 weeks after the second administration with 1 mL of the inactivated BoDV-1 strain V vaccine adjuvanted with mineral oil Montanide^TM^ ISA25. Five further rabbits were vaccinated accordingly with the nonadjuvanted vaccine adjusted to the same content of virus (10^7^ FFU). One control rabbit was carried along each group vaccinated with the corresponding vaccine formulation but without virus antigen. Experimental intracerebral infection was carried out two weeks after the last administration of the vaccine using strain V/B (homologous challenge infection, 10^2^ FFU). Body weights and symptoms were measured daily after infection. Antibodies (NT and IF) were determined after vaccination and infection in certain intervals.

#### 2.5.6. Vaccination of Horses with BoDV-1 Inactivated Vaccine “Dessau”

Trial VIII. These investigations were done on 1–10 years old female horses which were still held for the production of pregnant mare serum gonadotropin in Tornau in the early 1990s. Twelve horses from the stud were selected that were free of antibodies against BoDV-1 both by IF and NT. The horses (five in each group) were vaccinated s.c. with 1 mL of the inactivated vaccines ISA25-adjuvanted or nonadjuvanted at 10^7^ (lg 7) FFU before inactivation, again three weeks later, and a third time 16 weeks after third vaccination. Two horses served as controls and received the corresponding vaccines without antigen. Blood samples were drawn weekly. The antibody response (IF, NT) was followed up over 16 weeks.

#### 2.5.7. Vaccination of Horses with BoDV-1 Live Vaccine “Dessau”

Trial IX. In total, 38 horses were vaccinated in the field with the BoDV-1 live vaccine “Dessau”. The batches used were 186 03 89 and 194 03 90, produced in 1989 and 1990. Vaccinations were done in 1992 in Thuringia and southern Saxony-Anhalt when the vaccine was still authorized in the territory of the former German Democratic Republic. Thirty-one horses had been vaccinated prior to this vaccination, whereas 7 horses received their first administration of the vaccine. The age of the horses was between 2 and 15 years

### 2.6. PCR, Sequence Analysis and Phylogenetic Investigations

Six conventional RT-PCRs targeting N, N/X, X, P and subsequent sequencing were performed on both vaccine viruses as described [23]. For the vaccine strain “Dessau”, a 1824 bp-long sequence including N, N/X, X, P genes (GenBank acc. no. AY374519) had been established previously [23]. For the vaccine strain “Giessen”, a 3916 bp-long fragment including N, N/X, P, M and G genes (GenBank acc. no. DQ680832) had been sequenced in our previous study [91]. After confirmation of the previously established partial sequences, the total genome sequences of both vaccine strains were determined, using primers we had previously described [92]. ClustalW multiple sequence alignments of the complete coding BoDV-1 were conducted using BioEdit Sequence Alignment Editor Version 7.2.5.0. Phylogenetic trees based on the 8823-bp long sequences were created with the MEGA X program [93] by employing the Neighbor Joining method and p-distance algorithm with 1000 replicates of bootstrap resampling analysis. All bootstrap values <80 were omitted. Further alignments were done using Geneious Prime 2021.2.2 and amino acid compositions were evaluated using BioEdit 7.2.5.0.

## 3. Results

### 3.1. Genetic Characterization of Vaccine Viruses

**Figure 1:**
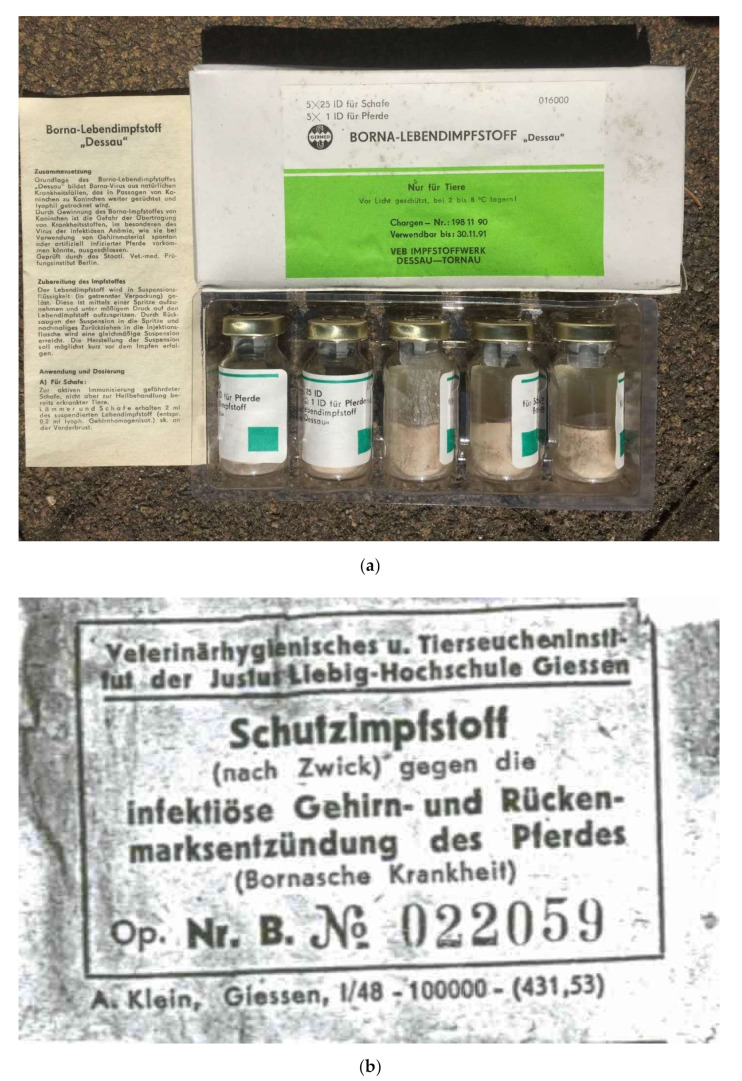
BoDV-1 vaccines used in Germany. (**a**) BD live virus vaccine “Dessau”, instructions for use (in German), package, and five vials containing lyophilized rabbit brain with BoDV-1 “Dessau” (cluster 3). Shown is the penultimate batch produced in November 1990, which was sequenced in this study (**b**) Label (in German) of the Zwick vaccine “Giessen” containing strain V from 1948, which was sequenced in this study (cluster 4).

We determined the full genomic sequences of the two vaccine viruses available in Germany, the East German BoDV-1 vaccine “Dessau” and the West German virus V Zwick “Giessen” (Figure 1).

#### 3.1.1. Vaccine Virus V Zwick “Giessen”

**Figure 2:**
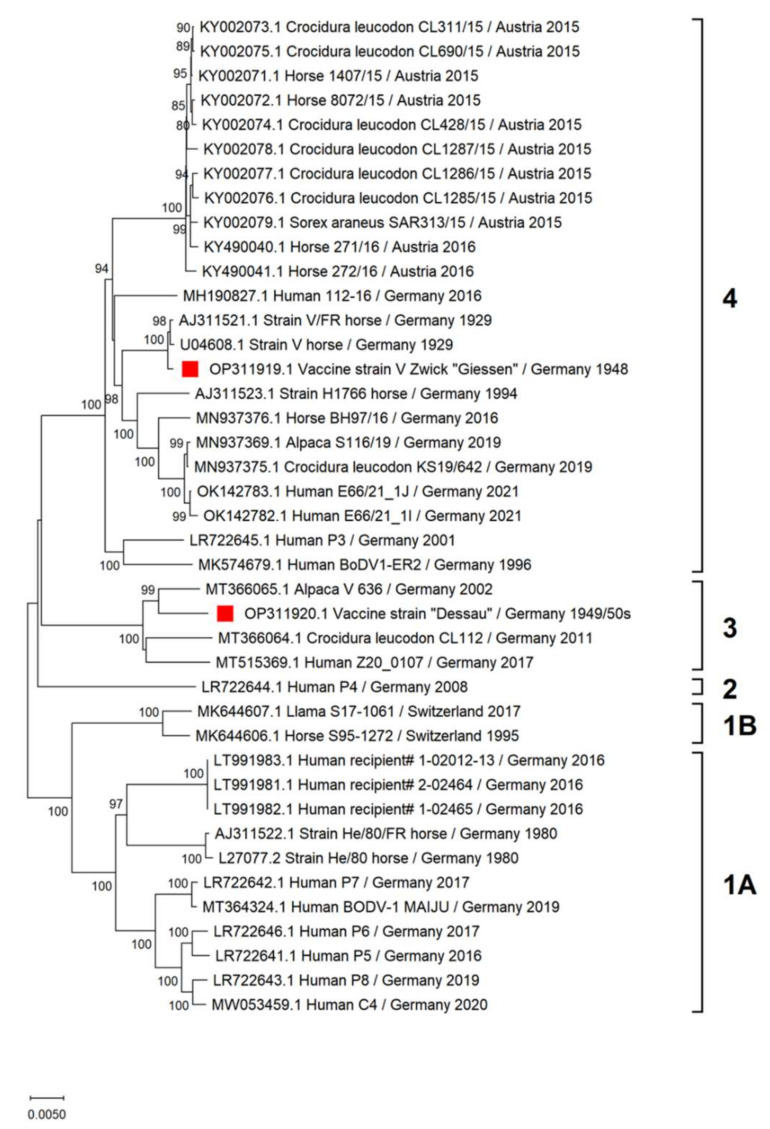
Phylogenetic analyses of the vaccine viruses Zwick “Giessen” and “Dessau” (red squares). The tree comprises 41 full genomes (8823 bp) of BoDV-1 (MEGA X, Neighbor-Joining method, p-distance model, pairwise deletion option, 1000 replicates, bootstrap values ≥ 80).

The genomic sequence established for the virus V Zwick “Giessen” (strain V/GZ, GenBank acc. no. OP311919) underwent phylogenetic analysis demonstrating that the Zwick “Giessen” vaccine virus belongs to BoDV-1 cluster 4 (Figure 2). When compared with 40 other complete BoDV-1 genomes, only a few amino acid substitutions were found in the strain V group comprising the Zwick “Giessen” virus of 1948 (V/GZ) and its derivatives, the Berlin (V/B) and Freiburg virus (V/FR) (Table 1). Additional amino acid alterations occurred during passaging from virus V Zwick “Giessen” (GZ) to “Berlin” (V/B) and “Freiburg” (V/FR), of which most were located in the L polymerase: 1× in P, 2× in G, 8× in L (Supplementary Material S4).

#### 3.1.2. Vaccine Virus “Dessau”

The established sequence was deposited under GenBank acc. no. OP311920. The vaccine virus “Dessau” belongs to BoDV-1 cluster 3 (Figure 2). This sequence is identical to the previously published partial BoDV-1 “Dessau” sequence of Kolodziejek et al., 1995 [23]. Compared with the 40 other complete BoDV-1 genomes, the “Dessau” sequence displayed an accumulation of amino acid differences in the M gene and N-terminal part of the G gene (Table 2; see Supplementary Material S4 for further information). A further sequence reported to originate from a BoDV-1 vaccine strain (MT374543; passage 64 of DessVac in OL-221 cells) is placed in cluster 4 but at another position than viruses of the strain V group or H1766 group (Supplementary Materials S4 and S5) and is, hence, unequivocally representing an independent virus isolate whose origin remains elusive [94,95].

#### 3.1.3. Virulence of Vaccine Virus “Dessau” in Rats

Both Wistar rat mothers of the litters inoculated with vaccine virus were infected by their offspring and succumbed to Borna disease; RT-PCR revealed presence of virus in the brain of these deceased animals. Of note, the clinical presentation of the rat mother infected with “Dessau” live vaccine virus resembled that of the rat mother infected with the BoDV-1 strain V/B; i.e., phenotype differences between the illness induced by vaccine vs. virus V/B could not be observed. Their litters did not develop clinical disease. However, presence of BoDV-1 was detected in multiple organs, which is a typical finding in persistently infected animals.

### 3.2. Inactivation Kinetics

**Figure 3:**
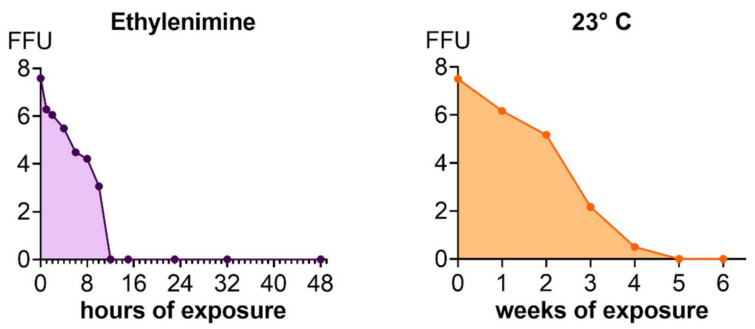
Inactivation kinetics of BoDV-1 “Dessau” during binary ethylenimine treatment (**left**) and storage at room temperature (**right**).

In order to prepare inactivated vaccines, inactivation kinetics were determined. Briefly, 12 h of treatment with binary ethylenimine led to BoDV-1 inactivation (Figure 3). Passaging of the inactivated material three times in YRB cells confirmed the inactivation as no foci were detected even at the lowest dilution. Storage at room temperature revealed complete inactivation of BoDV-1 after 5 weeks (Figure 3).

### 3.3. Immunological Aspects of Vaccination

#### 3.3.1. High Doses of Cell-Cultured Infectious Virus Protect from Disease

**Figure 4:**
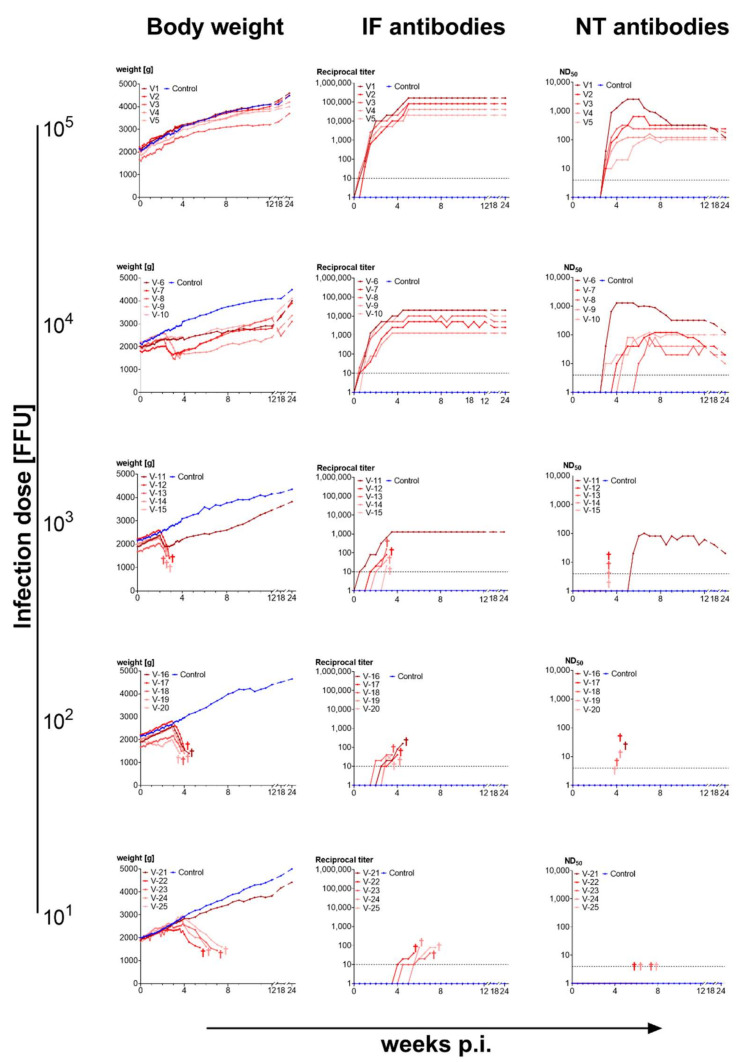
Titration of infectious dose of OL-cell-culture-derived BoDV-1 (virus V/B) in rabbits. Shown are the kinetics of body weights, immunofluorescent (IF) antibodies and neutralizing (NT) antibodies following infection challenge with 10^5^ FFU OL-derived BoDV-1, 10^4^ FFU OL-derived BoDV-1, 10^3^ FFU OL-derived BoDV-1, 10^2^ FFU OL-derived BoDV-1, and 10^1^ FFU OL-derived BoDV-1. Dashed lines, IF and NT assay limits of detection; †, animal euthanized before succumbing to lethal illness.

Rabbits infected with 10^5^ FFU BoDV-1 (strain V) grown on OL cells developed a strong and early response of antibodies detectable by both IF and NT assays and were clearly protected from disease upon infection. IF antibodies appeared between 3 and 7 days p.i., reached their highest levels between 3.5 and 5 weeks, and remained at this high level until 2 years p.i. NT antibodies appeared 2.5 to 3 weeks p.i., peaked between 4.5 to 7 weeks p.i. and decreased slightly thereafter. The rabbits displayed body weight gains, similar to those of the uninfected controls. No clinical symptoms were observed, even though a high virus dose had been administered intracerebrally (Figure 4).

Rabbits infected with 10^4^ FFU of cell-cultured virus had a short period of stagnation or even loss in body weights between 3–4 weeks after infection. They showed no central-nervous or other symptoms. All animals developed IF and NT antibodies. IF antibody kinetics were similar to that observed for the 10^5^ FFU dose but at lower level. NT antibodies appeared later in three rabbits (4–5.5 weeks p.i.), peaked at lower level in most of the individual rabbits but followed a similar course as those seen at infectious dose 10^5^ FFU (Figure 4). 

Rabbits infected with lower doses showed a more severe clinical course than those infected with higher doses: Of the five rabbits infected with 10^3^ FFU of OL-BoDV-1, only one survived. This rabbit showed a delayed development of IF and NT antibodies. The other four rabbits developed central-nervous symptoms three-four weeks after infection and were euthanized before their condition deteriorated. They showed low titres in IF but no NT antibodies (Figure 4). 

Among the five rabbits infected with 10^2^ FFU of OL-BoDV-1, all developed severe Borna disease (albeit with a later symptom onset than in the group infected with 10^3^ FFU BoDV-1) and had to be euthanized. They did not develop neutralizing antibodies (Figure 4). 

In the group infected with 10^1^ FFU of OL-derived BoDV-1 all but one rabbits succumbed to BD. The latter did not become infected most probably due to the dilution of the inoculum (no virus contained in the suspension injected by chance) (Figure 4). Thus, an infection dose of 10^2^ FFU BoDV-1 appeared to be the most reliable to induce BD.

**Figure 5:**
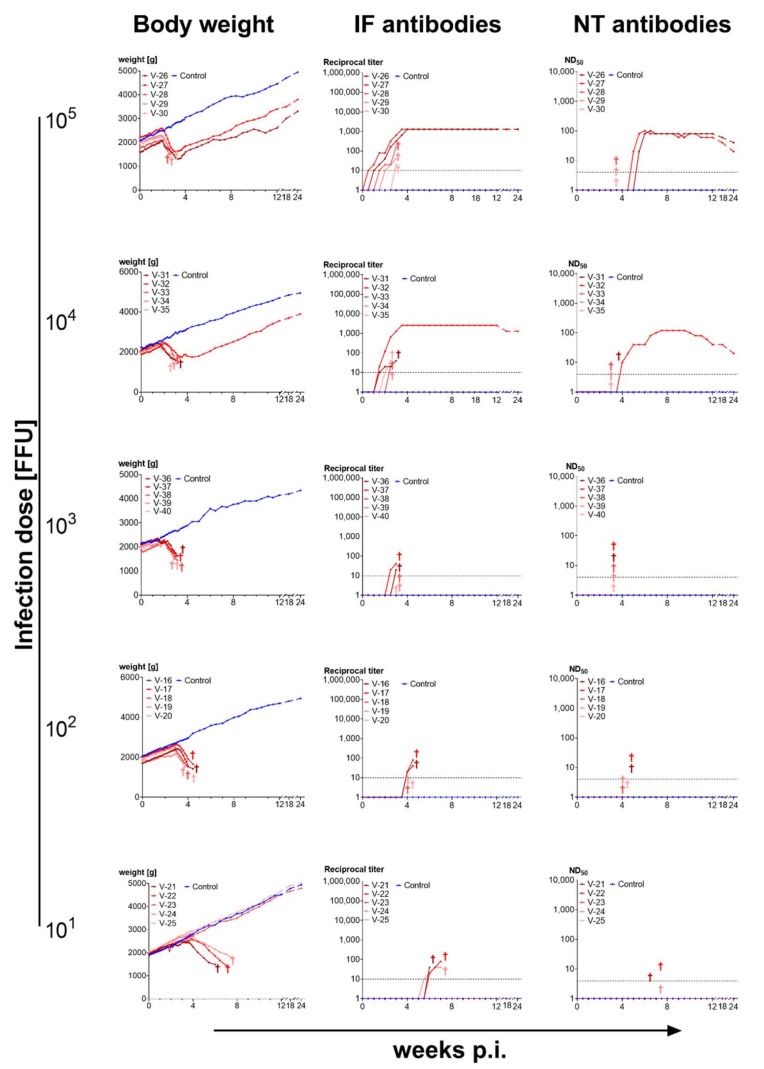
Titration of infectious dose of brain-derived BoDV-1 (strain V/B) in rabbits. Shown are the kinetics of body weight changes, immunofluorescent (IF) antibody titers and neutralizing (NT) antibody titers following infection challenge with 10^5^ FFU brain-derived BoDV-1, 10^4^ FFU brain-derived BoDV-1, 10^3^ brain-derived BoDV-1, 10^2^ FFU brain-derived BoDV-1, and 10^1^ FFU brain-derived BoDV-1. Dashed lines, IF and NT assay limits of detection; †, animal euthanized before succumbing to lethal illness.

The outcome in rabbits infected with different doses of brain-derived BoDV-1 differed remarkably from that observed in rabbits infected with cell-cultured virus: Even at the highest infection doses 3 of 5 rabbits succumbed to BD and the surviving rabbits showed a period of loss in body weights. The surviving animals developed neutralizing antibodies whereas the fatal cases did not (Figure 5).

Brain samples of the rabbits that succumbed to disease had strong RT-PCR viral signals, whereas brain samples of surviving rabbits euthanized at the end of the trial were PCR-negative for BoDV-1. In the five rabbits investigated for the presence of virus after high-dose infection (trial IV), positive signals by RT-PCR BoDV-1-were only found 1–3 weeks p.i. in the frontal cortex (near injection site) but not in other regions of the brain and not in organs outside the CNS (heart, liver, spleen, kidney, bladder, intestine).

After high numbers of BoDV-1 infected CL6 cells or high numbers of non-ultrasonicated OL/TL cells were injected into rabbits (trial III), protective effects were similar to those observed with high doses of virus obtained after ultrasonication of infected OL cells (data not shown). Similarly, injection of high doses of YRB cells infected with BoDV-1 protected infected rabbits, even though there was a delay in the induction of neutralizing antibodies, which was associated with a short period of weight loss in the rabbits (data not shown).

#### 3.3.2. Titration of Vaccine Doses

The BoDV-1 live vaccine “Dessau” was compared to an inactivated vaccine based on the “Dessau” strain.

**Figure 6:**
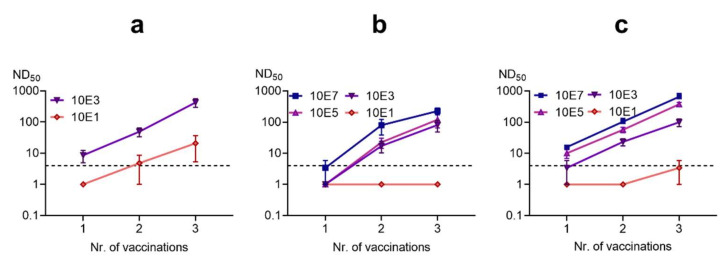
Titration of vaccine doses: Neutralizing antibody titers in vaccinated rabbits. BoDV-1 live vaccine “Dessau” containing 10^3^ FFU and 10^1^ FFU per dose (**a**) were compared with batches of ethylenimine-inactivated BoDV-1 “Dessau” vaccines containing either (**b**) no adjuvant and 10^7^, 10^5^, 10^3^, 10^1^ FFU BoDV-1 or (**c**) ISA25 (mineral oil-based o/w) adjuvant and 10^7^, 10^5^, 10^3^, 10^1^ FFU BoDV-1 before inactivation. Shown is the mean ± SEM of neutralizing antibody titers (ND_50_) in rabbits after the first, second (3 weeks after 1st) and third (16 weeks after 2nd) vaccine administration; titer determinations were based on BoDV-1 strain V/B. Dashed lines, NT assay limit of detection.

The BoDV-1 live vaccine was administered at the dose used in clinical practice (i.e., undiluted, 10^3^ FFU) and at 100-fold dilution of this material. A single dose of the undiluted vaccine induced neutralizing antibodies in three out of five rabbits. Administration of a second and third vaccine dose were followed by robust, consistent antibody responses (Figure 6). Of note, the 100-fold diluted live vaccine (10^1^ FFU dose) resulted in measurable (albeit low-level) NT antibody responses in two of five animals after three doses had been administered.

In contrast, the inactivated, unadjuvanted vaccine appeared considerably less immunogenic at comparable doses. Two inoculations with the 10^3^ FFU dose were required to induce NT antibodies in three animals. The third dose induced neutralizing titers in all animals, which were, however, an order of magnitude lower than those observed in animals immunized with the live vaccine. If the ISA25 adjuvant was used, the inactivated vaccine resulted in somewhat increased NT antibody responses, but not to the levels observed after live vaccine administration. In order to consistently induce high NT antibody titers in all animals, three high doses (10^7^ FFU before inactivation) of inactivated vaccine were required. At the high dose, addition of ISA25 resulted in NT antibody titers that were higher than those observed after administration of three 10^3^ FFU doses of the live vaccine “Dessau”.

#### 3.3.3. 90% of Rabbits Vaccinated with BoDV-1 Live Vaccine “Dessau” Were Protected from Disease after Challenge Infection

**Figure 7:**
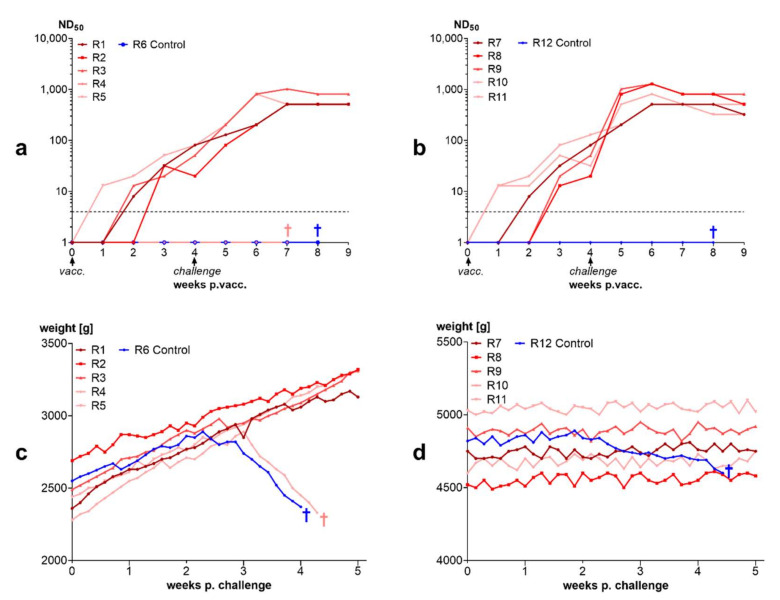
Neutralizing (NT) antibody titer kinetics in rabbits vaccinated with BoDV-1 live vaccine “Dessau” after (**a**) heterologous challenge infection with BoDV-1 V/B or (**b**) homologous challenge infection with the BoDV-1 vaccine virus “Dessau”. Body weight development after the respective challenge infections is shown in (**c**,**d**). Dashed lines, IF and NT assay limits of detection; †, animal euthanized before succumbing to lethal illness.

Rabbits vaccinated with a single dose of the BoDV-1 live vaccine “Dessau” underwent challenge infection, as did unvaccinated control rabbits. Whereas controls developed typical central-nervous symptoms, the majority of rabbits previously immunized with the live virus-vaccine did not develop any clinical symptoms (Figure 7).

Neutralizing antibodies in vaccinated rabbits increased slowly from week 1 after vaccination to weeks 3 and 4 after vaccination. One rabbit showed no detectable neutralizing antibodies at challenge infection. This rabbit developed BD. All other vaccinated rabbits responded to challenge with an increase in neutralizing antibodies within one week (Figure 7). IF antibodies showed a similar course (Supplementary Material S6). The two unvaccinated control rabbits succumbed to BD after challenge.

#### 3.3.4. Inactivated Vaccines Induce Antibodies in Rabbits and Protect from Disease

**Figure 8:**
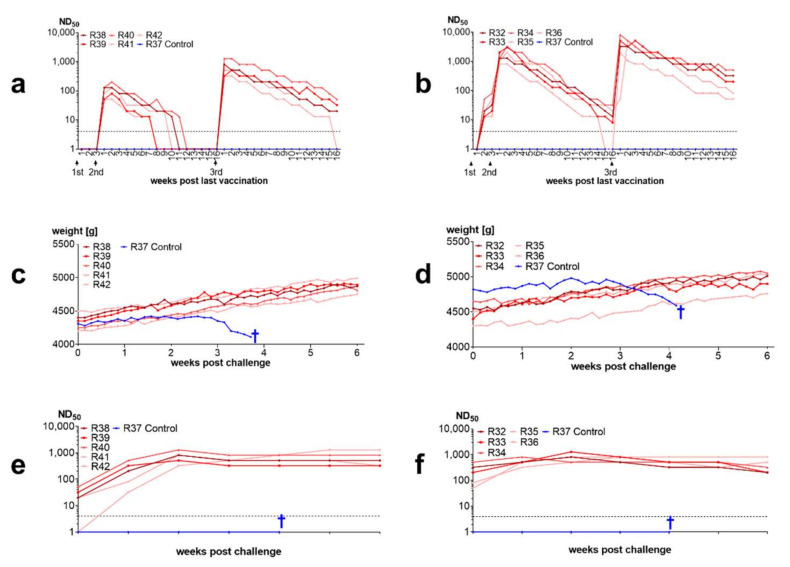
NT antibody kinetics in rabbits after vaccination with inactivated vaccines based on BoDV-1 strain V/B, following three doses of nonadjuvanted vaccine (**a**) or of ISA25-adjuvanted vaccine (**b**). Body weights after challenge with BoDV-1 V/B in animals vaccinated with nonadjuvanted inactivate (**c**) and in the animals vaccinated with ISA25-adjuvanted inactivated vaccine (**d**). NT antibody titers [ND_50_] in the nonadjuvanted group after challenge infection (**e**) and in the ISA25-adjuvanted group after challenge infection (**f**). Dashed lines, IF and NT assay limits of detection; †, animal euthanized before succumbing to lethal illness.

Threefold vaccination with an inactivate induced neutralizing antibodies in rabbits. There was almost no serological response to the first administration of the vaccine in rabbits immunized with the nonadjuvanted vaccine. Strong antibody increases were seen after the second dose and even higher increases were observed after the third dose. All vaccinated rabbits were protected from intracerebral challenge whereas unvaccinated rabbits developed BD. One week after challenge neutralizing antibody titers increased and remained at high level until the end of the trial (Figure 8).

#### 3.3.5. Inactivated Vaccines Stimulate Antibodies in Horses

**Figure 9:**
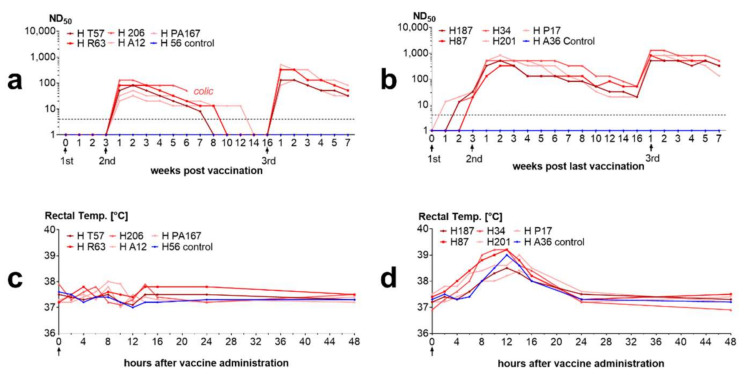
NT antibody kinetics in horses after vaccination with inactivated vaccines based on BoDV-1 “Dessau”, following three applications of the nonadjuvanted vaccine (**a**), or ISA25-adjuvanted vaccine (**b**). Rectal temperatures after the first vaccine administration in horses vaccinated with the nonadjuvanted inactivated vaccine (**c**) and in the group vaccinated with the ISA25-adjuvanted inactivated vaccine (**d**). One horse developed colic and had to be taken out of the trial. Dashed lines, NT assay limit of detection.

Similar to rabbits, horses developed antibodies after vaccination with inactivated vaccines. Antibodies were higher in the horses vaccinated with the adjuvanted vaccine. Horses vaccinated with the adjuvanted vaccine displayed a temporary increase in body temperature within the first 24 h after each vaccination. This increase in body temperatures was also seen in the control horses vaccinated with adjuvanted control vaccine without BoDV-1 antigen, but not in the horses vaccinated with the unadjuvanted vaccine (Figure 9). Furthermore, horses vaccinated with the ISA25 vaccine displayed local swellings at the site of the injection.

#### 3.3.6. The BoDV-1 Live Vaccine “Dessau” Induces Neutralizing Antibodies in Horses

Twenty-two of the 31 repeatedly vaccinated horses (71%) displayed a neutralizing antibody response to vaccination (Table 3). Neutralizing antibodies were already detectable 1 week after administration of the vaccine (pvacc). Neutralizing antibody titers peaked 1–3 weeks pvacc and decreased thereafter in most of the horses. Four months pvacc only 11 horses (35%) still had antibodies at detectable levels. Five of the 31 horses displayed neutralizing antibodies prior to vaccination, indicating that these antibodies had persisted in 16% of the horses for approximately one year. No adverse events were observed in the horses and none of the horses developed disease within the observation period of 17–22 weeks. One horse developed a high neutralizing antibody response. This horse was eight years old and had been vaccinated every year (i.e., this was the seventh administration of the vaccine). It had a neutralizing antibody titer before vaccination (80 ND_50_), which remained at the same level after vaccination. Four weeks after administration of the vaccine, the neutralizing antibody titer increased to 300 ND_50_. It reached 640 ND_50_ six weeks later and stayed at this level until the end of the trial.

Five of the 7 horses vaccinated for the first time (71%) and, hence, without pre-existing immunity developed antibodies after live virus vaccination, but it took longer before antibodies were first detectable and neutralizing antibodies could only be detected for a short time period (Table 4). No adverse events were observed and no horse developed disease within the 17 weeks observation period. The data indicate that 30% of the horses did not respond to vaccination with antibodies.

## 4. Discussion

### 4.1. Sequence Characterization of BoDV-1 Vaccine Viruses

We have established the genomic sequences of two BoDV-1 vaccine viruses used for live horse and sheep vaccination in Germany in the 20th century. The vaccine virus “Dessau” was originally isolated from a horse which had succumbed to BD according to interviews with former staff of Impfstoffwerk Dessau-Tornau. The exact regional location of the horse is unknown. However, it is well-known that BoDV-1 sequences originating from the same geographic region display high levels of similarity over decades [23]. The “Dessau” sequence clusters within clade 3, which includes viruses from Saxony and central Saxony-Anhalt [23,96]. Currently, no BoDV-1 sequences are available from the name-giving region (Borna), where the disease became extinct probably due to the effect of strong environmental pollution caused by lignite mining and coal-fired power stations on reservoir or transmitting species [33]. However, the “Dessau” virus may indeed originate from the Borna region. This interpretation is based on the observation that the most closely related sequence was established from an alpaca in the Saxon Switzerland East Ore Mountains district of Saxony, southeast of Borna [97] and that the original brain material may have been provided by the pathology institute of the Leipzig veterinary faculty which received a lot of submissions from the area around Borna.

The vaccine virus “Dessau” has been derived from a naturally circulating virus that underwent over 100 (between 128 and 199), the exact number being unknown) rabbit passages [58]. This original “Dessau” virus strain is not available anymore, which complicates the determination of potential attenuation markers. Comparison of the “Dessau” vaccine virus with other natural viruses may provide hints to the changes that occurred during passaging. The “Dessau” virus displays 15 unique amino acid substitutions which are not present in other BoDV-1. Whereas the amino acid composition in the N, P, and L genes do not exceed the rate of unique amino acid alterations seen in other BoDV-1 and could therefore simply represent the sequence of the original virus, the high number of amino acid alterations in the N-terminal part of G and M is striking. These alterations were probably caused by the high number of rabbit passages, which has also led to a reduced incubation period [58]. Thus, the N-terminal part of G, the envelope glycoprotein, and M may have enabled adaptation to rabbits. Because passaging was done from brain to brain, i.e., within the CNS and the N-terminal part of G (G-N) is only present in virions budding from cells, this adaptation in G suggests that particle formation is relevant in brain tissue. 

We found no virulence differences between strain “Dessau” and strain V/B in infected Wistar rats, indicating that the rabbit passages may not have induced attenuation in the sense of reducing or abolishing BoDV-1 virulence in species other than rabbits. These findings are in contrast to those of Schulz et al., who described a loss of virulence in horses and sheep [58]. While Schulz et al., do mention that infection with the vaccine virus did not induce disease in horses and sheep, the data they present is based exclusively on subcutaneous infections with high doses of the vaccine virus, which did not result in disease. It should be noted that the subcutaneous infection most probably induced immunity and can therefore not be regarded as proof for a loss of virulence.

The Zwick vaccine virus “Giessen” analyzed in this study originates from 1948. Given the time period of its origin, passaging was most likely done in rabbits only, presumably at less than 50 times. Zwick named BoDV-1 strains numerically; strain 5 or V was first mentioned in reference [71]. In reference [98], Zwick et al., used strains III and IV. From this, it can be concluded that strain V might have been isolated in the late 1920s. While the putative geographic origin of the strain V virus has previously been reported to be the Hannover (Lower Saxony) region [23], this area was not given as region of origin by Zwick et al. [71]; thus it might also be possible that the virus originated from Thuringia or Hesse or other geographical regions of BoDV-1 cluster 4. Strain V was brought from Giessen to Berlin in 1978. Prior to sequencing of the Berlin variant (V/B), this virus underwent passages in rats and in mice. The Freiburg variant of strain V (V/FR), shipped from Berlin to Freiburg in 1995, underwent additional passages in mice [70]. These viruses comprise the strain V group. They show only a limited number of unique mutations differentiating them from other viruses, which do not exceed those seen in other BoDV-1. This might potentially be explained by the species switch during passaging, which might have precluded the adaptations in the G and M proteins observed for the “Dessau” vaccine virus. Within the strain V group, mutations accumulated in the L polymerase during passaging, especially in strain V/FR which may reflect the frequent laboratory use of this virus.

Despite the widespread use of strain V-based vaccines in Bavaria, clade 4 BoDV-1 are rare in Bavaria. A natural population of this cluster is present only in Franconia [23]. Therefore, it is reasonable to assume that circulation of the vaccine virus does not occur naturally. Similarly, in Eastern Germany, where the BoDV-1 live vaccine “Dessau” was used, identical viruses have not been identified. Especially in Thuringia, where cluster 4 is present, clade 3 viruses were not identified [23].

### 4.2. Immunogenicity of BoDV-1 Depends on Infection Dose and Virus Source

The data presented here demonstrate that BoDV-1 is immunogenic. High doses of cell-culture-derived BoDV-1, injected intracerebrally (i.c.), consistently induced a strong immune response associated with virus clearance in rabbits. Remarkably, these rabbits remained asymptomatic, indicating a lack of pathogenic processes and T-cell dependent immunopathology. By contrast, high doses of BoDV-1 derived from the brain of infected animals, injected i.c., induced BD in the majority of experimentally infected rabbits. The few surviving animals displayed a slower neutralizing antibody response than rabbits infected i.c. with high doses of cell-culture-derived BoDV-1. Similarly, low doses of cell-cultured BoDV-1, injected i.c., induced BD.

Our finding that high-dose infection with cell-culture derived BoDV-1 is protective in rabbits dates to the 1990s and is consistent with data obtained in experimentally infected rats [36,48]. These investigations revealed a short period of viral replication following injection of high doses of cell-culture-derived BoDV-1, with a peak around 12 dpi and viral clearance around 18 dpi [36].

Our observation that high dose-infection with cell-culture-derived BoDV-1 did not result in disease while low-dose infection with cell-culture-derived BoDV-1, as well as high-dose infection with brain-derived BoDV-1 did, could be explained by the presence of immunogenic (and thus protective) viral components that are diluted too far in low-dose infection and expressed at low levels in brain tissue. This data is in line with findings of Stitz et al. [99] and Rall et al. [52], also indicating that the induction of protective immune responses requires a strong immunogenic stimulus.

Protective immunity against Bornavirus disease has been shown to be mediated by T-cell responses directed against the N protein [36] in a process involving CD8^+^ T-cell-dependent antiviral mechanisms [39]. Thus, a plausible explanation for the observed phenomenon is that high infection doses function as strong immunogenic stimulus, inducing a rapid, potently antiviral T-cell response; while low infection doses might not suffice as immunogenic stimulus for an antiviral T-cell response, thus resulting in intracerebral viral spread to a point where T-cell responses contribute to clinical disease. Of note, T-cell responses have been shown to be time-dependent, with high activity in the early stages of infection and signs of tolerance/exhaustion during the later infection stages [100].

We did not investigate T-cell responses in our studies, but we did measure IF antibody levels. While IF-antibodies may target all BoDV-1 proteins [101], previous studies imply that they recognize predominantly the soluble protein including P and N [66], the latter is key to the protective as well as the pathogenic effects of T-cell activity [39,51]. Thus, IF-antibody levels might serve as a rough proxy for anti-N antibody levels, which in turn might be regarded as a rough proxy for the presence of T-cell activating stimuli. We observed rapid, high-level IF antibody responses in those animals that did not develop disease. Antibodies against N and P are non-neutralizing. 

The early presence of neutralizing antibodies represented another pronounced difference between protected and unprotected rabbits in our study: Neutralizing antibodies were absent in animals that died, indicating that they may contribute substantially to the role of T-cell mediated immune responses in the inhibition of viral spread, thereby supporting viral clearance. Neutralizing antibodies peaked 4 weeks after high-dose infection; around this time, viral antigens disappeared in the infected animals, as shown for rabbits in our trial (IV), but also for rats in other investigations [36,48]. Similarly, animals that had displayed weight loss or stagnation following infection (10^4^ FFU, trial IIA) began to regain their body weights as NT antibody titers rose. This underscores the notion that neutralizing antibodies might represent another crucial component in protection after immunization. Neutralizing antibodies are induced by the two BoDV-1 glycoproteins (glycoprotein G or gp94 and matrix protein M or gp18) [99,102,103]. While neutralizing antibodies targeting M did not induce viral clearance in BoDV-1-infected Lewis rats (albeit at a late infection stage) [102], an influence on virus dissemination was demonstrated for neutralizing antibodies targeting G in vivo [73]. Following entry into the central nervous system, BoDV-1 mainly replicates via RNPs, which do not contain glycoprotein G [104]; glycoprotein G appears to be required only at the cell-to-cell interfaces, enabling budding and uptake of virions into new cells to permit further transport of the virus [105], although this has been controversially discussed [106]. Given the extremely low amount of glycoprotein G at neuronal synapses and junctions, virus propagation within the central nervous system might not be dampened by the humoral immune response. The expression of G differs between cells, also in the brain [101,105]. In contrast, higher amounts of cell-associated virus are generated in cell culture [107]. The amounts of glycoprotein G produced in the brain are probably very low compared to those of BoDV-1-infected cell cultures, in line with the comparatively low neutralizing antibody titers observed in our experiment [trial IIB], and potentially explaining the insufficient protection by high doses of brain-derived virus in the infectious dose titration experiments.

In previously published experimental trials, not all animals developed disease consistently [43,44,108,109,110]. This might be explained by administration of different infection doses, in particular because efficient BoDV-1 titration techniques have only become available after antibody staining techniques were developed in the 1970s [45,111,112].

After high-dose infection with BoDV-1, IF antibodies were detected earlier (one week p.i.) than NT antibodies (2 to 3 weeks p.i.). This delayed appearance of NT antibodies could be due to a lower amount of glycoprotein G than other proteins in the inoculum [11,73]. Moreover, the downregulation of BoDV-1 G in infected cells, their limited proteolytic processing and the protection of antigenic epitopes on G by host-identical glycans are considered key mechanisms used by this pathogen to evade host immune responses and thus establish viral persistence [113]; these mechanisms may also play a role in the delayed appearance of anti-G-antibodies in infected animals. Indeed, the efficient induction of NT antibody responses seen at 2–3 weeks p.i. might be the result of BoDV-1 replication; i.e., inoculation primed the immune response while subsequent replication of the inoculated virus in boosted the immune response, thus inducing relevant NT antibody levels at a later timepoint. Similar delays in the NT antibody response were seen after live vaccination in horses, who received a single vaccine dose and developed low titers of neutralizing antibodies mostly 3–5 weeks after vaccination (this paper, see Section 3.3.6).

IF antibodies were found in 78% of horses with natural BD shortly before death but only 9% of them displayed neutralizing antibodies [33]. This indicates that low expression of the G and M protein, enabling neutralization escape, may be a key factor protecting this virus from early elimination by the immune response.

### 4.3. Inactivated BoDV-1 Vaccines Are Capable of Inducing Protection against Clinical Disease

These data led us to hypothesize that inactivated vaccines based on purified virions may be able to protect from BD because they contain more glycoprotein to stimulate immune reactions. Inactivated vaccines (carbolic acid, glycerine, formaldehyde, 50 °C temperature inactivation) based on brain suspensions of infected rabbits failed in the investigations of Zwick, Seifried and Witte [61], whereas live vaccine was successful [114]. Thus, most subsequent vaccine development efforts focused on live vaccines. Nevertheless, some attempts to use formaldehyde-inactivated vaccines were successful [47,115,116,117]. A potential explanation for the early failure of the first inactivated vaccines [61] could be that the brain suspensions used were diluted before inactivation, which decreased the already low content of glycoprotein below protective levels. Another is the vaccination scheme: Most approaches followed a single vaccination strategy; only the partially successful approach of Nicolau and Galloway employed more than one vaccination [47]. Of note, Hameed et al., investigated an inactivated vaccine based on avian bornavirus in cockatiels [38]. They applied a threefold vaccination scheme followed by a booster with recombinant N. At the subsequent challenge infection, most of the vaccinated birds were protected against clinical disease, although they did developed persistent subclinical infection in the absence of disease.

### 4.4. Antibody Kinetics and Immunity after Live Vaccination

After vaccination with the BoDV-1 live vaccine “Dessau”, 70% of horses (trial IX) and 90% of rabbits (trial VI) developed neutralizing antibodies. All rabbits with NT antibodies were protected against clinical disease after challenge. In horses, antibodies peaked late (between 3–5 weeks after first vaccination). A potential explanation is that local replication of the virus was required to boost the immune response induced by the first vaccine dose (prime). The subsequent decline and disappearance of neutralizing antibodies indicates virus elimination because otherwise antibodies would have been expected to increase further; and none of the horses developed disease which would have been the result had the virus reached the CNS. The investigations of Lüthgen (1977) showed that BoDV-1 inoculated subcutaneously into rabbits reached the brain in 50% of the animals [118]. Additionally, investigations of Matthias (1958) indicate a replication of BoDV-1 at the injection site [43,44]: Administering high amounts of apathogenic cocci immediately after s.c. or i.n. administration of BoDV-1 increased the percentage of rabbits and horses developing clinical Borna disease. Matthias’ findings could potentially indicate that replication outside nerval tissues generally induces protective immune responses, but that these are very weak and can be suppressed by parallel stimulation of the immune system with other agents. Thus, although live vaccination is protective, this vaccination mode may bear two inherent risks: (i) an inadvertent injection into neural tissue could take place in some cases, (ii) inadequate immune responses (in the course of immune suppression or parallel immune stimulation by other agents) may enable virus replication at injection site and further propagation of virus to neural tissue. Both processes could induce clinical BD.

Most repeatedly vaccinated horses showed a peak in neutralizing antibody titers within 1–2 weeks after repeat vaccination which hints to a booster reaction (trial IX). A few horses showed late and low neutralizing antibody titers similar to those observed for first-vaccinated horses, indicating that the first time their immune system recognized the neutralizing antigen was at this (repeat) vaccine administration. 30% of the repeatedly vaccinated horses did not react at all, indicating that the neutralizing antigen was neither detected at the first nor at the repeat vaccine administration, suggesting that the virus did not replicate at all at injection site.

As shown for the horses vaccinated for the first time, one administration of the vaccine is not sufficient because it induces either low antibody titers, which do not persist, or no antibodies at all. A second administration of the live vaccine, within a month after the first vaccine dose, would have been beneficial. As no booster vaccination was applied, not all horses responded with neutralizing antibodies to vaccination and duration of immunity was short. This explains why not all horses vaccinated with BoDV-1 live vaccine were protected in clinical and experimental studies. Lüthgen (1977) could detect IF antibodies only in a low number of horses but he investigated horses 8 and 16 weeks after vaccination [118], a period when antibodies are already after the period of decline. The low titers also may indicate that the antigen content of the Oberschleissheim vaccine was low. He could not find neutralizing antibodies in subcutaneously immunized rabbits, most probably because the sensitivity of his NT assay was low due to the high amount of virus used for neutralization (10^5^ FFU). Additionally, other investigations using the complement fixation assay could only rarely detect antibodies after vaccination [109,119,120]. Thus, our data is in line with published data showing that live vaccination protects, but not in all cases: Field trials with live vaccines showed a reduction in Borna cases in vaccinated horses in most of the trials [37,46,59,121,122,123] (summarized by Danner [Table 23 page 227: 3.8% Borna cases in not vaccinated horses vs. 0.6% Borna cases in vaccinated horses on average). Investigations in sheep reflected even better results: 0.5–2% cases in unvaccinated sheep vs. 0.005% cases in vaccinated sheep [58].

The protective effects of antibodies observed in the field trial in horses were also confirmed by experimental infection of rabbits: 90% of the rabbits immunized with live BoDV-1 developed antibodies and were protected at challenge infection. The lack of antibody induction in one rabbit may be explained by failure of replication of the virus at injection site. In this context, it should be noted that the percentage of animals responding to vaccination with antibody production was higher in rabbits than in horses. There might be species-specific differences in BoDV-1 local replication after subcutaneous infection. This could be a reason why sheep display better vaccination responses than horses despite administration of lower doses [58]. It has been demonstrated that after BoDV-1 has entered the CNS, further viral spread cannot be stopped by neutralizing antibodies [73]. Peripheral priming by vaccination can support quick induction of neutralizing antibodies as shown for one rabbit in our studies of inactivated vaccines. This rabbit (R42, see Figure 8) had no measurable antibodies at challenge infection but responded with quick production of neutralizing antibodies after challenge infection and did not develop disease.

### 4.5. Historic Perspective

Use of inactivated vaccines was abandoned in the 1920s because the first inactivated BoDV-1 vaccines did not induce effective immune protection. The studies reported here provide an explanation for this lack in protection. As shown for the unadjuvanted vaccine, there was no seroresponse after single-dose vaccination in rabbits as well as in horses. Four factors may have contributed to the failure of inactivated vaccines in the 1920s: (i) inadequate vaccination scheme focusing on single dose administration instead of three dose administration (second three weeks after first; third four months after second; thereafter yearly is recommended), (ii) use of brain material instead of cell-cultured virus (the techniques to produce the latter were not available at this time), (iii) administration of low antigen doses and (iv) no use of adjuvants. Other vaccination trials based on cell-cultured BoDV-1 in which vaccination of rabbits with inactivated BoDV-1 laboratory batches failed were reported by Danner [32]. Kurt Danner provided no details about his vaccination experiments, indicating that the results were preliminary but he came to similar conclusions, i.e., that high amounts of antigen were necessary for vaccination [32]. New production techniques were available in the 1980s after the investigation of Georg Pauli which enabled for the first time the production of high numbers of virions in cell culture [49]. Interest in BoDV-1 vaccine development has faded due to decreasing numbers of BD cases in horses since the 1960s [124]. Thus, the BoDV-1 live vaccine “Dessau” remains the single authorized vaccine so far. Within the entire period of vaccination with BoDV-1 live vaccines in horses and sheep of 64 years this vaccine was in use for 43 years.

### 4.6. Limitations and Future Perspectives

Our studies have several limitations:

First, we did not investigate whether vaccination protects against infection. Importantly, Hameed et al., (2018) found that vaccination of cockatiels with an inactivated vaccine as well with a recombinant PaBV-4 N protein induced protection from clinical disease, similar to our results; however, it did not prevent persistent, subclinical infection following PaBV-2 challenge. Persistent subclinical infection is not desirable because the healthy-appearing infected birds may transmit virus to others in the flock [38]. The mechanisms underlying the observed protection against clinical disease are unknown. Hameed at al. hypothesized that the vaccine induced a non-pathogenic type 2 immune response; in line with this hypothesis, they found that animals treated with Cyclosporine A prior to challenge were protected from clinical disease [38]. This is in line with the results of Hausmann et al., (2005), who concluded that CD8^+^ T-cell-dependent non-cytolytic immune responses may be important for the immunological control of bornaviruses [39]. It might also be possible that vaccination could induce an exhaustion of the antiviral T-cell response, similar to that observed by Narayan et al., in bornavirus-infected rats [100], thus reducing clinical illness in vaccinated animals. There were certain differences between the inactivated vaccine used by Hameed at al. [38] and the inactivated vaccines used in our study, which may affect the comparability of the results: (i) use of different production cell lines (avian in Hameed et al.’s and mammalian in our approach) and (ii) use of sonicated infected cells in Hameed et al.’s and cell-free virus in our approach. Of note, Hameed et al., did not investigate Bornavirus-neutralizing antibodies, which have not been detected in birds so far [125], potentially due to the low glycoprotein expression in avian cells [101]. In contrast to the investigations of Hameed et al., (2018) our approach induced neutralizing antibodies. As long as neutralizing antibodies are detectable after vaccination, this status of neutralizing activity is similar to that reported by Furrer et al., (2001) in Lewis rats prophylactically treated with anti-G antibodies three days and one day before infection and then every 7 days [73]; this approach prevented infection after intrafootpad inoculation before BoDV-1 entered the CNS [73]. In contrast to treatment with antibodies, vaccination induces active antibody responses.

Vaccination can be applied only prophylactically. Hameed et al., (2018) showed that vaccination after infection does not prevent disease [38]. It is most probably also not possible to eliminate bornavirus in persistently infected carriers by vaccination. Rall et al., (2019) immunized persistently infected cockatiels three times with mixtures of rMVA/rNDV/N/P vaccines but could not eliminate the infection despite induction of humoral immune responses [52]. An elimination of persistent infection by vaccine-induced T cells appears highly unlikely if not impossible, given the tolerance against the immunodominant T-cell epitope associated with BoDV-1 persistence. However, the impact of G-protein-containing vaccines on persistent infection should still be thoroughly investigated, because these vaccines might reduce the amount of viral shedding in persistent infection by inducing neutralizing antibodies: Stitz et al., (1998) were able to restrict the presence of BoDV-1 to the CNS in rats by passive transfer of neutralizing antibodies even in rats infected as newborns clearly demonstrating that dissemination of virus can be blocked by neutralizing antibodies [99].

Other effects of neutralizing antibodies have been observed: For example, splenectomized rabbits infected intraperitoneally developed high titres in neutralizing antibodies which lasted over months [66]. A multifocal retinopathy was seen in these rabbits but healed when neutralizing antibodies appeared. Moreover, no virus could be isolated from the brain of these rabbits in the presence of neutralizing antibodies [66]. Rats showed weight gains after neutralizing antibodies appeared [1]. Infected rats with neutralizing antibodies very often displayed an obesity syndrome [1]. After experimental mouse-adaptation of BoDV-1, neutralizing antibodies were also observed in this species [1,126]. In vitro assays showed that the virus spread was prevented by neutralizing sera and infection was restricted to the first infected cell [66,99]. Thus, neutralizing antibodies mainly prevent virus spread. In combination with T-cell activity there could be a synergistic effect, as observed in the high-dose infection trials.

Second, similar to Hameed et al., (2018) we did not analyze T-cell responses. Further studies are needed to elucidate if and to what extent T-cell responses are involved in the immune response to live or inactivated BoDV-1 vaccines [38].

Third, our immunity studies were of limited duration. For the high-dose ISA25-adjuvanted inactivate vaccine, our data indicate protection from week 1 after administration of the second vaccine dose until week 16 after the third vaccine dose, but antibody data indicate that protection lasts longer. Future studies should include studies on the longterm-duration of these and other successful vaccination approaches, such as the vector-based vaccination strategies (Supplementary Material S7). Interestingly, protection from infection 30 days after high-dose infection has previously been demonstrated in outbred CD rats by Oldach et al., (1995) [48].

Fourth the mineral oil adjuvant showed side effects (temporary fever induction in horses). Thus, future vaccination approaches may benefit from the use of less reactogenic adjuvants that still enhance vaccine immunogenicity [127].

Fifth, the number of control animals was restricted to one per experiment in our vaccination studies. We followed this approach for ethical reasons, given that the infection dose applied (10^2^ FFU) was already known to reliably induce clinical disease, based on our previous studies.

Sixth, we did not investigate antibodies in the brain but only in sera. IF antibodies in the brain and cerebrospinal fluid (CSF) are produced locally (intrathecally) after intracerebral infection, with titers differing only slightly from those detected in serum [118,128], whereas NT antibody titers in CSF are usually higher than in serum [129]. It is interesting that we found increased serum antibody titers after intrathecal antibody generation, given that the blood-brain- barrier has been shown to be intact in BoDV-1-infection [128]. A possible explanation for the serum antibody response induced after intracerebral infection could be that the blood-brain-barrier is crossed by immune cells including T-cells [130,131,132,133] as well as B-cells for which migration via diapedesis has been demonstrated in some investigations [134,135,136]. Thus, the fast emergence of serum antibodies in rabbits after high-dose intracerebral infection indicates that immune responses in the brain also impact responses outside of this site. Vice versa, peripheral immunogenic stimuli, in particular vaccination, may also have a protective influence on CNS infection (i.e., peripheral priming may influence antibody reaction patterns in the brain), given that neutralizing antiviral antibodies present in the brain, play an important role for the elimination of other viruses from the CNS [137,138].

The investigations show that vaccination against BoDV-1 is possible when using sufficient doses of antigen and vaccination schemes which include three vaccinations at least. The rapid development of mRNA technologies for vaccination in recent years [139,140,141] offers the possibility to develop economic and highly protective vaccines against BoDV-1 in the near future. Vaccines based on a combination of G and N might be the best approach for future bornavirus vaccine developments.

## 5. Conclusions

The most effective experimental way to protect from Borna disease so far is the intracerebral injection of high doses of bornaviruses. In this setting, high antigen doses stimulate a strong T-cell response which eliminates the virus and induce early neutralizing antibodies, which block virus dissemination thereby supporting the antiviral action of T-cells. Other successful approaches have been vaccinations with vector-based vaccines containing N, which were associated with preventing virus infection when administered at high doses. This approach most probably does not induce long-lasting protection against infection, because antiviral T cell responses are downregulated over time [100]. Prevention from disease has been demonstrated for live and inactivated vaccines, but the underlying protective immunological mechanisms are still unknown. For horses, it has been shown in a limited number of challenge trials that approximately 2/3 were protected from disease even, when challenge was performed several months after vaccination [30,46]. This protection rate corresponds to the seroconversion rate in neutralizing antibodies and potentially, these antibodies might indeed be involved in protection. Because neutralizing antibodies can block the distribution of the virus, this mechanism can be very effective in preventing infection at entry. Because the natural bornavirus transmission route is unknown, it is also important to investigate the processes that occur when the virus does reach the CNS—as modelled in i.c. infections. In this scenario, disease can be prevented by vaccination with live and inactivated vaccines, but the protective immunological mechanisms have not been elucidated and it is still unknown whether the virus persists under vaccine-induced immunological control in mammals or not. The induction of neutralizing antibodies by inactivated vaccines in mammals is a remarkable difference to the investigations in birds where no neutralizing antibodies were detected. These antibodies may contribute to the reduction or even prevention of viral shedding [99]. Three vaccine administrations, with the third administration not too close to the second (e.g., four months after), can induce longer persistence of neutralizing antibodies. Future vaccination approaches may benefit from the use of vaccines that contain G-protein in addition to N-protein. The limited success of BoDV-1 vaccines in the past was also due to suboptimal vaccine compositions and vaccination schemes, which can now be improved. In the course of the development of new vaccination technologies further progress in vaccination against BD is to be expected. The development of new vaccination technologies, including vector-based recombinant or mRNA vaccine-based techniques, needs to take into account processes that were also observed in our studies, such as the decline of immune responses after antigen contact and the requirement for several antigen contacts in order to induce potent, broad immune responses, as shown recently for the SARS-CoV-2 vaccines [142,143,144,145,146,147,148,149,150,151,152].

## Figures and Tables

**Table 1:** Overview of amino acid substitutions present only in strain V viruses, but not in other BoDV-1.

Number of Mutations Protein	1	2	3	4	5	6	7	8
N	-	-	-	-	-	-	-	-
P	I34V	-	-	-	-	-	-	-
M	E108D	-	-	-	-	-	-	-
G	A17V	-	-	-	-	-	-	-
L	S134F	T158A	T1430A	I1431V	-	-	-	-

**Table 2:** Overview of amino acid substitutions present only in BoDV-1 “Dessau”, but not in other BoDV-1.

Number of Mutations Protein	1	2	3	4	5	6	7	8
N	P30S	-	-	-	-	-	-	-
P	-	-	-	-	-	-	-	-
M	S71F	V73A	T86I	-	-	-	-	-
G	R44K	Q145R	L186V	N192K	Y236H	K245E	T251I	A288V
L	V382I	I543V	V1545A	-	-	-	-	-

**Table 3:** Neutralizing antibody titers after vaccination with BoDV-1 live vaccine “Dessau” in horses already vaccinated in the years prior.

Horse	Weeks Post Vaccination										
	0	1	2	3	4	5	8	10	13	17	22
I	<4 ^1^	**75**	- ^2^	**80**	**20**	-	-	<4	-	<4	-
II	<4	**17**	-	**40**	**35**	-	-	**4**	-	**4**	-
III	<4	**10**	-	-	**16**	-	-	<4	-	<4	-
IV	<4	**8**	-	-	**32**	-	-	**4**	-	<4	-
V	<4	**15**		**15**	**8**	-	-	**8**	-	**6**	-
VI	<4	<4	-	<4	<4	-	-	<4	-	<4	-
VII	<4	-	-	**25**	**11**	-	-	<4	-	<4	-
VIII	<4	-	-	**30**	**28**	-	-	**4**	-	**6**	-
IX	<4	**80**	-	**40**	-	-	-	**32**	-	**20**	-
X	<4	**16**	-	**80**	-	-	-		-	<4	-
XI	8	**32**	-	**32**	-	-	-	**32**	-	**24**	-
XII	<4	<4	-	**4**	-	-	-	**4**	-	<4	-
XIII	**16**	**32**	35	-	-	-	-	-	-	16	-
XIV	**16**	**16**	32	**20**	-	-	-	-	-	<4	-
XV	<4	**31**	18	**18**	-	-	-	-	-	<4	-
XVI	<4	<4	-	-	-	-	-	-	-	-	-
XVII	<4	**30**	**80**	**16**	**18**	-	-	-	-	<4	-
XVIII	<4	**32**	**85**	**35**	**20**	-	-	-	-	**10**	-
XIX	**4**	**80**	**32**	**21**	-	8	-	-	-	**8**	-
XX	<4	**15**	**27**	**30**	-	8	-	-	-	**8**	-
XXI	<4	**15**	**31**	**17**	-	<4	-	-	-	<4	-
XXII	<4	<4	<4	-	<4	-	<4	-	<4	-	<4
XXIII	<4	<4	<4	-	<4	-	<4	-	<4	-	<4
XXIV	<4	<4	<4	-	<4	-	<4	-	<4	-	<4
XXV	<4	<4	<4	-	<4	-	<4	-	<4	-	<4
XXVI	<4	<4	<4	-	**10**	-	<4	-	<4	-	<4
XXVII	<4	<4	<4	-	<4	-	<4	-	<4	-	<4
XXVIII	**80**	**80**	**80**	-	**300**	-	**520**	-	**640**	-	**640**
XXiX	<4	80	80	-	80	-	16	-	4	-	<4
XXX	<4	<4	<4	-	<4	-	<4	-	<4	-	<4
XXXI	<4	**160**	**80**	-	**80**	-	**8**	-	**8**	-	**8**

^1^ <4, detection limit; ^2^ -, no blood sample taken, bold, neutralizing activity in serum, grey, duration of antibody positivity.

**Table 4:** Neutralizing antibody titers after first vaccination with BoDV-1 live vaccine “Dessau” in horses.

Horse	Weeks Post Vaccination										
	0	1	2	3	4	5	8	10	13	17	22
XXXII	<4 ^1^	<4	- ^2^	-	-	-	-	<4	-	<4	-
XXXIII	<4	<4	-	<4	-	**4**	-	<4	-	<4	-
XXXIV	<4	<4	**8**	**60**	-	**40**	-	<4	-	<4	-
XXXV	<4	<4	<4-	<4	-	**16**	-	<4	-	<4	-
XXXVI	<4	<4	<4	<4	-	<4	-	<4	-	<4	-
XXXVII	<4	**4**	**4**	**4**	-	**30**	-	-	-	<4	-
XXXVIII	<4	<4	-	32		-	-	<4	-	<4	-

^1^ <4, detection limit; ^2^ -, no blood sample taken, bold, neutralizing activity in serum, grey, duration of antibody positivity.

## Data Availability

Not applicable.

## Supplementary Materials

The following supporting information can be downloaded at: https://www.mdpi.com/article/10.3390/v14122706/s1, Supplementary Material S1: Overview of history of vaccination against Borna disease. Supplementary Material S2: History of vaccine viruses “Dessau” and V (Zwick “Giessen”). Supplementary Material S3: Overview of infection trials. Supplementary Material S4: Genetic analyses of BoDV-1 vaccine viruses. Supplementary Material S5: Phylogenetic analysis of sequence information of the “Dessau” virus differing from the sequence established here. Supplementary Material S6: Immunofluorescence antibody studies. Supplementary Material S7: Overview of successful vaccine approaches.
