# Supplementary material for: Vaccination against Borna Disease: Overview, Vaccine Virus Characterization and Investigation of Live and Inactivated Vaccines
Source: Viruses. 2022 Dec 2;14(12):2706. doi: 10.3390/v14122706 (PMC9788498; doi:10.3390/v14122706)
Supplement: Supplementary file 1 [file viruses-14-02706-s001.zip › Supplementary Material S1.pdf]

Dürrwald R, Kolodziejek J, Oh D-Y, Herzog S, Liebermann H, Osterrieder N, Nowotny N.
Vaccination against Borna disease: overview, characterization of vaccine viruses and Investigation of live and inactivated vaccines

Supplementary Material S1.

Supplementary Table S1.1. History of vaccination against Borna disease.

**Year(s)**	**Finding**	**Consequences**	**References**
1924/25	Experimental transmission of Borna disease from horse to rabbits by using ultrafiltrated brain homogenate	Viral etiology proven as basis for immunization	[1, 2]
1925	First immunisation of rabbits s.c, i.p.	Rabbits protected	[2]
1926	First immunisation of a horse (3x s.c. live vaccination with rabbit brain)	Horse was protected against i.c. challenge 6 months after last vaccination	[3]
1928	First vaccinations of horses in the field	Introduction of vaccination of horses against BD in Germany	**[4, 5]**
1920s/1930s	Investigations of inactivated vaccines	Failure or<br>partial protection	**[2, 6, 7]**<br>**[8]**
1930s	Vaccination with live vaccines based on BoDV-1 horse brain	Wide use of vaccination in southern Germany	**[9, 10]**
1931	Vaccination with dried live virus	Later uses for vaccination in the central German endemic territory	**[11]**
1940s	Transmission of infectious anemia virus due to vaccination with dried live virus	Live vaccination with vaccines based on horse brain abolished	**[12]**
1947-49	Development of BD live virus vaccine “Dessau”	Used for vaccination of horses and sheep for more than 40 years	**[13, 14]**
1950s	Simultaneous infection with bacteria increased probability of development of BD after s.c., i.n. and i.c. BoDV-1 infection	Exposure to strong other antigens may overlap BoDV-1 antigen and forces immune responses into another direction, thus allowing sufficient replication of the virus to reach CNS	**[6, 15]**

Supplementary Table S1.1 continued. History of vaccination against Borna disease.

**Year(s)**	**Finding**	**Consequences**	**References**
1970s	Fluorescent techniques allow the detection of BoDV-1 antigen in infected cells	Cultivation of BoDV-1 in cell culture possible	**[16]**
1984	Increase of virus yields from persistently infected cells by chemicals and salts	Allowed the characterization of the virus particle ten years later	**[17]**
1992	Live vaccination in Germany abolished	No vaccinations against Borna disease since 1992	**[18]**
1994	First sequences of BoDV-1 established	Classification into a new family of ssnRNA viruses	**[19]**
1990s	Administration of high doses of cell-cultured BoDV-1 induces immune protection	First indication that immunogenic components of BoDV-1 are lacking at low dose infection	**[20, 21]**
1990s/2000s	Cytotoxic T cells directed against the BoDV-1 nucleoprotein (N) are the cause of immunopathology	Pathogenesis solved	**[22-26]**
2000s	Priming with poxvirus-vector N vaccine enhances disease	Vaccination against N may cause side effects	**[27]**
2000s	Poxvirus-vector vaccines directed against N protect after multiple immunizations	Vaccination based on vector vaccines is possible	**[28, 29]**
2001	Identification of the cytotoxic H-2K^k-restricted T-cell epitope	The T-cell epitope immunodominant in mice was characterized	**[30, 31]**
2017/2018	Investigation of vector-based vaccines against avian bornaviruses	Protection by a combination of vector-based vaccines (NDV, MVA) using prime and two boosts at high doses	**[32, 33]**
2018	Investigation of inactivated vaccines and vaccines based on recombinant protein without vector	Clinical protection but not protection from infection	**[34]**

[1] Details see Supplementary material S2, s.c., subcutaneous; i.n., intranasal; i.c., intracerebral

References

Dürrwald R, Kolodziejek J, Oh D-Y, Herzog S, Liebermann H, Osterrieder N, Nowotny N. Vaccination against Borna disease: overview, characterization of vaccine viruses and Investigation of live and inactivated vaccines

1. Zwick W, Seifried O. Uebertragbarkeit der seuchenhaften Gehirn- und Rückenmarksenrzündung des Pferdes (Borna'schen Krankheit) auf kleine Versuchstiere (Kaninchen). *Berl Tierärztl Wochenschr* 1925; **41**: 129-32.
2. Zwick W, Seifried O, Witte J. Experimentelle Untersuchungen über die seuchenhafte Gehirn- und Rückenmarksentzündung der Pferde (bornasche Krankheit). *Z Infektionskrh Haustiere* 1927; **30**: 42-136.
3. Zwick W, Seifried O, Witte J. Weitere Untersuchungen über die seuchenhafte Gehirn-Rückenmarksentzündung der Pferde (Bornasche Krankheit). *Z Infektionskr Haustiere* 1928; **32**: 150-79.
4. Zwick W. Heutiger Stand der Forschungen über die Bornasche Krankheit. *Berl Tierärztl Wochenschr* 1931; **47**: 797-8.
5. Steinbrück. Ueber die bornasche Krankheit der Einhufer, ihre Beziehungen zur Viehversicherung und Versuche zu ihrer Bekämpfung im Regierungsbezirk Merseburg. *Berl Tierärztl Wochenschr* 1931; **47**: 793-8.
6. Matthias D. Zur Epidemiologie der Bornaschen Krankheit. *Arch Exp Veterinärmed* 1955; **9**: 824-43.
7. Danner K. Borna-Virus und Borna-Infektionen. Vom Miasma zum Modell. Stuttgart: Enke 1982.
8. Nicolau S, Galloway IA. L'encéphalo-myélte enzootique experimentale (maladie de Borna). *Ann Inst Pasteur* 1930; **45**: 457-523.
9. Gminder A. Versuche über die Immunisierung gegen die infektiöse Gehirnrückenmarksentzündung (Bornasche Krankheit oder Kopfkrankheit) der Pferde in Württemberg. *Berl Tierärztl Wochenschr* 1934; **12**: 203-5.
10. Ernst W. Neuere Arbeiten über Encephalitiden bei Tieren. *Ergeb Hyg Bakteriol Immunitätsforsch Exp Therapie* 1931; **12**: 1-14.
11. Zwick W, Witte J. Über die Widerstandsfähigkeit des Virus der Bornaschen Krankheit gegen Trocknung und über Schutzimpfungsversuche mit getrockneter virushaltiger Gehirnsubstanz. *Berl Tierärztl Wochenschr* 1931; **47**: 33-5.
12. Fortner J. Die ansteckende Blutarmut der Einhufer in den Impfstoffwerken. *Berl Münch Tierärztl Wochenschr* 1948; **61**: 49-53.
13. Möhlmann H, Maas A. Wertigkeitsprüfung des Borna-Trockenimpfstoffes "Dessau" bei Pferden unter den Verhältnissen der Praxis. *Arch Exp Veterinarmed* 1960; **14**: 1267-80.
14. Schulz JA, Müller H, Lippmann R. Untersuchungen zur Prophylaxe der Bornaschen Krankheit bei Schafen mittels aktiver Immunisierung. *Arch Exp Veterinarmed* 1968; **22**: 571-83.
15. Matthias D. Weitere Untersuchungen zur Bornaschen Krankheit der Pferde und Schafe. *Arch Exp Veterinärmed* 1958; **12**: 920-47.
16. Mayr A, Danner K. Production of Borna virus in tissue culture. *Proc Soc Exp Biol Med* 1972; **140**: 511-5.
17. Pauli G, Ludwig H. Increase of virus yields and releases of Borna disease virus from persistently infected cells. *Virus Res* 1985; **2**: 29-33.
18. Dürrwald R. Die natürliche Borna-Virus-Infektion der Einhufer und Schafe: Untersuchungen zur Epidemiologie, zu neueren diagnostischen Methoden (ELISA, PCR) und zur Antikörperkinetik bei Pferden nach Vakzination mit Lebendimpfstoff.: Freie Universität Berlin, Germany; 1993.
19. Briese T, Schneemann A, Lewis AJ et al. Genomic organization of Borna disease virus. *Proc Natl Acad Sci U S A* 1994; **91**: 4362-6.
20. Oldach D, Zink MC, Pyper JM et al. Induction of protection against Borna disease by inoculation with high-dose-attenuated Borna disease virus. *Virology* 1995; **206**: 426-34.
21. Furrer E, Bilzer T, Stitz L, Planz O. High-dose Borna disease virus infection induces a nucleoprotein-specific cytotoxic T-lymphocyte response and prevention of immunopathology. *J Virol* 2001; **75**: 11700-8.

22. Richt JA, Stitz L, Wekerle H, Rott R. Borna disease, a progressive meningoencephalomyelitis as a model for CD4+ T cell-mediated immunopathology in the brain. *J Exp Med* 1989; **170**: 1045-50.
23. Richt J, Stitz L, Deschl U et al. Borna disease virus-induced meningoencephalomyelitis caused by a virus-specific CD4+ T cell-mediated immune reaction. *J Gen Virol* 1990; **71 (Pt 11)**: 2565-73.
24. Planz O, Bilzer T, Stitz L. Immunopathogenic role of T-cell subsets in Borna disease virus-induced progressive encephalitis. *J Virol* 1995; **69**: 896-903.
25. Planz O, Stitz L. Borna disease virus nucleoprotein (p40) is a major target for CD8(+)-T-cell-mediated immune response. *J Virol* 1999; **73**: 1715-8.
26. Hausmann J, Schamel K, Staeheli P. CD8(+) T lymphocytes mediate Borna disease virus-induced immunopathology independently of perforin. *J Virol* 2001; **75**: 10460-6.
27. Lewis AJ, Whitton JL, Hatalski CG et al. Effect of immune priming on Borna disease. *J Virol* 1999; **73**: 2541-6.
28. Henkel M, Planz O, Fischer T et al. Prevention of virus persistence and protection against immunopathology after Borna disease virus infection of the brain by a novel Orf virus recombinant. *J Virol* 2005; **79**: 314-25.
29. Hausmann J, Baur K, Engelhardt KR et al. Vaccine-induced protection against Borna disease in wild-type and perforin-deficient mice. *J Gen Virol* 2005; **86**: 399-403.
30. Schamel K, Staeheli P, Hausmann J. Identification of the immunodominant H-2K(k)-restricted cytotoxic T-cell epitope in the Borna disease virus nucleoprotein. *J Virol* 2001; **75**: 8579-88.
31. Richter K, Baur K, Ackermann A et al. Pathogenic potential of borna disease virus lacking the immunodominant CD8 T-cell epitope. *J Virol* 2007; **81**: 11187-94.
32. Runge S, Olbert M, Herden C et al. Viral vector vaccines protect cockatiels from inflammatory lesions after heterologous parrot bornavirus 2 challenge infection. *Vaccine* 2017; **35**: 557-63.
33. Rall I, Amann R, Malberg S et al. Recombinant Modified Vaccinia Virus Ankara (MVA) Vaccines Efficiently Protect Cockatiels Against Parrot Bornavirus Infection and Proventricular Dilatation Disease. *Viruses* 2019; **11**.
34. Hameed SS, Guo J, Tizard I et al. Studies on immunity and immunopathogenesis of parrot bornaviral disease in cockatiels. *Virology* 2018; **515**: 81-91.
