# Supplementary material for: Vaccination against Borna Disease: Overview, Vaccine Virus Characterization and Investigation of Live and Inactivated Vaccines
Source: Viruses. 2022 Dec 2;14(12):2706. doi: 10.3390/v14122706 (PMC9788498; doi:10.3390/v14122706)
Supplement: Supplementary file 1 [file viruses-14-02706-s001.zip › Supplementary material S2.pdf]

Dürrwald R, Kolodziejek J, Oh D-Y, Herzog S, Liebermann H, Osterrieder N, Nowotny N. Vaccination against Borna disease: overview, vaccine virus characterization and Investigation of live and inactivated vaccines

Supplementary material S2

Supplementary Table S2.1. History of the Borna disease live vaccine "Dessau".

Year(s)	Event
1947	The production of the vaccine Borna-Schutzimpfstoff "Dessau" started in the Asid-Serum-Institut Dessau; the vaccine was based on dried brain suspension of infected rabbits; base of the production in the years 1947-1949 was a BoDV-1 from sheep; the vaccine dose for one horse was 0.1 g dried brain suspension which was administered s.c. (also at the Isle of Riems 4500 vaccine doses Borna-Schutzimpfstoff were produced in 1947, 3800 doses for Saxony, 700 doses for Thuringia).
1949	A virus obtained from a horse which succumbed to BD was used as vaccine virus. According to personal communications there might have been some changes in the material (all from horses) used for production in the early 1950s but from then onward the same seed virus was used which was further passaged over the years.
1951/52	Vaccination challenge trials in Dessau show: 0.1 g, 0.2 g and 0.3 g dried brain suspension are insufficient for protection 7 months after vaccination; 2 of 3 horses which had been vaccinated with 0.5 g dried vaccine were protected againt i.c. challenge 7 months after vaccination; the third horse died during the trial due to another condition; the unvaccinated control horse succumbed to BD → the vaccination dose was increased 5-fold to 0.5 g (1 rabbit brain yielded two vaccine doses for horses) [1]. Vials were evacuated after lyophilisation which further increased shelf life.
1956	Passaging procedure in rabbits was changed: 18 days in rabbits (from passage 98 onward 15 days and from passage 128 onward 12 and 10 days, respectively; in 1967 the 128th passage was established; see also: [2] and [3]). The optimal virus harvest was determined: 3-6 days before end of incubation period. Infection of rabbits in the beginnings were done via trepanation; later injection was done via the Foramen occipitale without destruction of tissue.
1956/1957	Field trials on 2290 horses in 37 local communities of endemic areas (3990 vaccinations were carried out) compared with 2472 not vaccinated horses in 35 local communities of endemic areas → no significant differences between the groups [1].
1964	From production batch 58 onward direct lyophilisation in the vials; a solution containing 20% alumiumhydroxide was used as solvent because before there had been difficulties to resolve the dried brain material.<br>The vaccine contained 0.015 g dried brain suspension for each sheep until November 1963 which had to be resolved in 1.65 mL physiological NaCl solution; since 1964 the content was increased to 0.02 g lyophilised brain suspension per sheep which had to be resolved in 2 mL 20% aluminiumhydroxide suspension.
1960-1967	Investigations in 35 sheep herds: 77357 vaccinations, only 4 vaccinated sheep succumbed to Borna disease, significant reduction in losses in comparison to not vaccinated sheep [2].
1987-1991	Investigations in order to establish production in cell culture; a permanent cell line from Newborn Rabbit Brain (NRB) was established which accidentally got contaminated with the vaccine virus. The cell bank at the Friedrich-Loefller-Institute, Isle of Riems, was provided with this cell line.
1992	Authorization of the vaccine ceased. No further vaccinations against BD were conducted in Germany for meanwhile over 30 years.

Supplementary Table S2.2. History of the BoDV-1 strain V group.

Year(s)	Event
1920s	Wilhelm Zwick isolated bornaviruses (BoDV-1) from horses and named them them numerically; virus 5 or V was first mentioned in a publication in 1937[4]
1930s – 1980s	This virus was used as vaccine virus.
1951/52	Erhard Nitzschke in Gießen adapted this virus to rats and detected for the first time immunotolerance [5, 6].
1970s till date	Hanns Ludwig conducted several groundbreaking investigations using this virus (summarized in [7, 8]).
1978	Hanns Ludwig brought the virus from Giessen to Berlin [9]
1990	The first BoDV-1 cDNA clones were isolated and characterized from this virus in the USA [10].
1994	The first complete genome was established [11] together with virus He/80 (Herzog/80) [12].
1995	The virus was shipped from Berlin to Freiburg.
2002	A whole genomic sequence from the Freiburg virus was established [13].
1990s till date	The virus is available in many laboratories worldwide and several sequences were established from these viruses which form the strain V group.

References

1. Möhlmann H, Maas A. Wertigkeitsprüfung des Borna-Trockenimpfstoffes "Dessau" bei Pferden unter den Verhältnissen der Praxis. *Arch Exp Veterinarmed* 1960; **14**: 1267-80.
2. Schulz JA, Müller H, Lippmann R. Untersuchungen zur Prophylaxe der Bornaschen Krankheit bei Schafen mittels aktiver Immunisierung. *Arch Exp Veterinarmed* 1968; **22**: 571-83.
3. Möhlmann H. 10 Jahre Forschungsinstitut für Impfstoffe Dessau. *Arch Exp Veterinarmed* 1965; **19**: 253-60.
4. Zwick W, Witte J, Schwarzmaier E. Zur Frage der Pluralität des Bornavirus. *Z Infektionskr Haustiere* 1937; **51**: 261-7.
5. Nitzschke E. Variation beim Virus der Bornaschen Krankheit (infektiöse Encephalomyelitis der Pferde und Schafe) durch Rattenpassagen. *VIIth International Congress for Microbiology*. Stockholm 1958:275-6.
6. Nitzschke E. Untersuchungen über die experimentelle Bornavirus-Infektion bei der Ratte. *Zentralbl Veterinärmed* 1963; **B 10**: 470-527.
7. Ludwig H, Bode L, Gosztonyi G. Borna disease: a persistent virus infection of the central nervous system. *Prog Med Virol* 1988; **35**: 107-51.
8. Ludwig H, Furuya K, Bode L et al. Biology and neurobiology of Borna disease viruses (BDV), defined by antibodies, neutralizability and their pathogenic potential. *Arch Virol Suppl* 1993; **7**: 111-33.
9. Dieckhöfer R. Epidemiologische Untersuchungen zur equinen BDV-Infektion, der Bornaschen Krankheit beim Pferd, der Therapie und die dazugehörige aktuelle Gesetzessituation in Deutschland.: Freie Universität Berlin, Germany; 2006.
10. Lipkin WI, Travis GH, Carbone KM, Wilson MC. Isolation and characterization of Borna disease agent cDNA clones. *Proc Natl Acad Sci U S A* 1990; **87**: 4184-8.
11. Briese T, Schneemann A, Lewis AJ et al. Genomic organization of Borna disease virus. *Proc Natl Acad Sci U S A* 1994; **91**: 4362-6.
12. Cubitt B, Oldstone C, de la Torre JC. Sequence and genome organization of Borna disease virus. *J Virol* 1994; **68**: 1382-96.
13. Pleschka S, Staeheli P, Kolodziejek J et al. Conservation of coding potential and terminal sequences in four different isolates of Borna disease virus. *J Gen Virol* 2001; **82**: 2681-90.
