# Supplementary material for: Vaccination against Borna Disease: Overview, Vaccine Virus Characterization and Investigation of Live and Inactivated Vaccines
Source: Viruses. 2022 Dec 2;14(12):2706. doi: 10.3390/v14122706 (PMC9788498; doi:10.3390/v14122706)
Supplement: Supplementary file 1 [file viruses-14-02706-s001.zip › Supplementary material S6.pdf]

Dürrwald R, Kolodziejek J, Oh D-Y, Herzog S, Liebermann H, Osterrieder N, Nowotny N. Vaccination against Borna disease: overview, vaccine virus characterization and investigation of live and inactivated vaccines

Supplementary material S6: Immunofluorescent antibodies

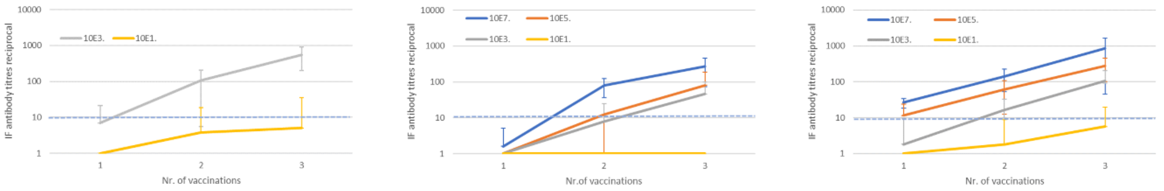

(a) (b) (c)

Supplemetary Figure S6.1: Figure 6 extended. IF antibodies. Titration of the vaccine doses. BoDV-1 live vaccine "Dessau" containing lg3 (e) and lg1 FFU (h) per dose were compared with batches of inactivated vaccines of BoDV-1 "Dessau" which contained eiter no adjuvant and lg7 (a), lg5 (c), lg3 (f), and lg1 (i) or ISA25 (mineral oil-based) adjuvant and lg7 (b), lg5 (d), lg3 (g), and lg1 (f) before inactivation with ethylenimine. Shown are the neutralizing antibody titres (ND_{50}) in rabbits after first, second (3 weeks after 1st) and third administration (16 weeks after 2nd) of the vaccine: rhombs – geometric mean with standard deviation, dots – individual antibody titres (for serological investigations virus V was used); (a) one rabbit died from coccidiosis two weeks after first vaccine administration; therefore, only data for four rabbits are provided in this group after the following administrations of the vaccine. Dashed line, detection limit.

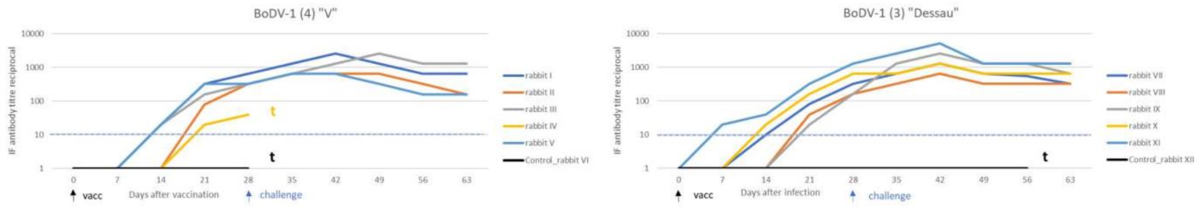

(a) (b)

Supplementaty Figure S6.2: Figure 7 extended. IF antibody kinetics after vaccination of rabbits with Borna live vaccine "Dessau" and after heterologous (a) and homologous challenge infection (b). Dashed line, detection limit.

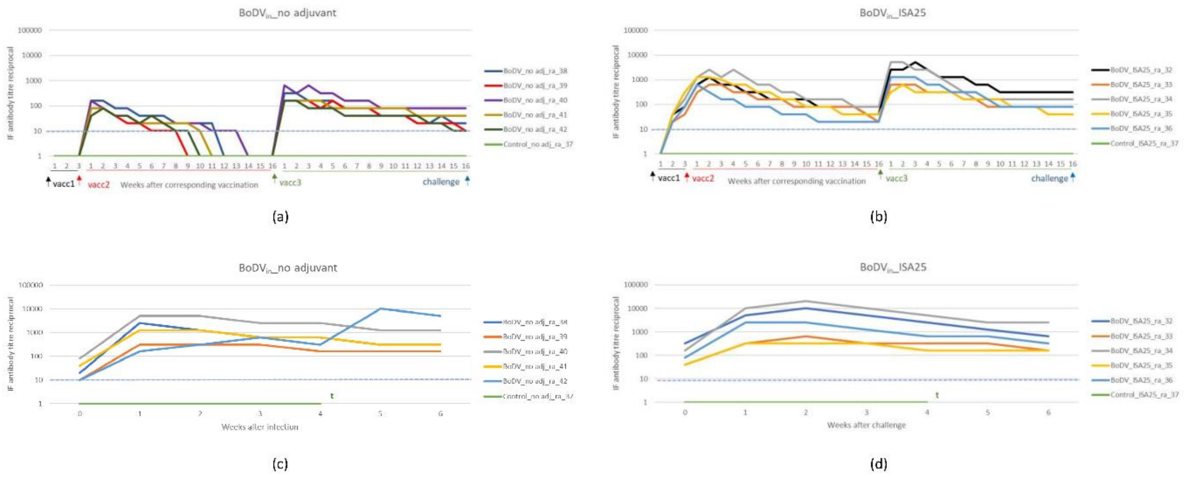

Supplementary Figure S6.3: Figure 8 extended. IF antibody kinetics after vaccination of rabbits with inactivated vaccines based on BoDV-1 "Dessau" after threefold vaccination with not adjuvanted vaccine (a), with ISA25-adjuvanted vaccine (b), IF antibodies in the not adjuvanted group after challenge and in the ISA25-adjuvanted group (c) after challenge (d). Dashed line, detection limit.

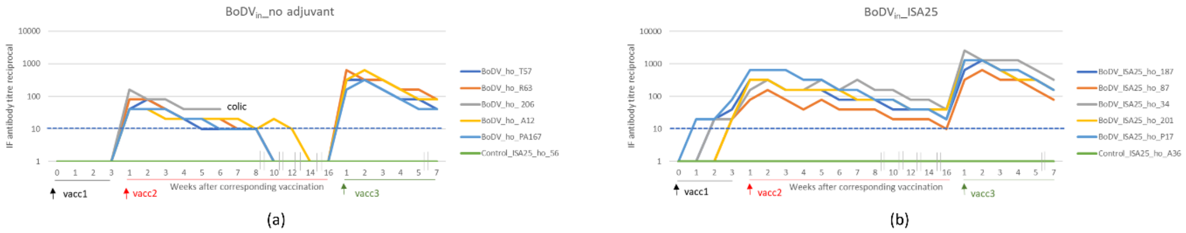

Supplementary Figure S6.4: Figure 9 extended IF antibody kinetics after vaccination of horses with inactivated vaccines based on BoDV-1 "Dessau" after threefold vaccination with not adjuvanted vaccine (a), with ISA25-adjuvanted vaccine (b), one horse developed colic and had to be taken out of the trial (a). Dashed line, detection limit.

Supplementary Table S6.1: Table 3 extended. Immunofluorescent antibodies after vaccination with BoDV-1 live vaccine "Dessau" in horses which had been already vaccinated previously.

Horse	Weeks post vaccination										
	0	1	2	3	4	5	8	10	13	17	22
I	<10[1]	320	-[2]	640	160	-	-	80	-	40	-
II	<10	160	-	160	160	-	-	160	-	80	-
III	<10	80	-	-	80	-	-	40	-	<10	-
IV	<10	160	-	-	80	-	-	40	-	20	-
V	<10	80		80	80	-	-	80	-	40	-
VI	<10	20	-	<10	<10	-	-	<10	-	<10	-
VII	<10	-	-	160	160	-	-	40	-	<10	-
VIII	<10	-	-	320	320	-	-	80	-	80	-
IX	20	640	-	320	-	-	-	320	-	160	-
X	<10	160	-	80	-	-	-		-	<10	-
XI	20	320	-	320	-	-	-	320	-	320	-
XII	10	160	-	80	-	-	-	80	-	10	-
XIII	40	320	320	-	-	-	-	-	-	40	-
XIV	40	160	320	160	-	-	-	-	-	20	-
XV	10	320	160	160	-	-	-	-	-	<10	-
XVI	<10	80	-	-	-	-	-	-	-	-	-
XVII	<10	320	80	160	80	-	-	-	-	40	-
XVIII	20	320	640	320	320	-	-	-	-	80	-
XIX	20	320	320	320	-	160	-	-	-	80	-
XX	<10	160	320	320	-	80	-	-	-	80	-
XXI	<10	160	320	160	-	80	-	-	-	40	-
XXII	<10	40	40	-	20	-	<10	<10	<10	<10	<10
XXIII	<10	20	20	-	10	-	<10	<10	<10	<10	<10
XXIV	<10	80	40	-	20	-	20	<10	<10	<10	<10
XXV	<10	<10	<10	-	<10	-	<10	<10	<10	<10	<10
XXVI	20	80	80	-	160	-	40	40	10	<10	<10
XXVII	<10	<10	<10	-	<10	-	<10	<10	<10	<10	<10
XXVIII	320	320	320	-	1280	-	5120	-	5120	-	2560
XXiX	<10	320	320	-	320	-	160	-	80	-	40
XXX	<10	<10	<10	-	<10	-	<10	-	<10	-	<10
XXXI	40	640	160	-	160	-	160	-	160	-	160

[1] <10, detection limit; [2] - , no blood sample taken

Supplementary Table S6.2: Table 4 extended. Immunofluorescent antibodies after first vaccination with BoDV-1 live vaccine "Dessau" in horses.

Horse	Weeks post vaccination										
	0	1	2	3	4	5	8	10	13	17	22
XXXII	<10[1]	<10	-[2]	-	-	-	-	<10	-	<10	-
XXXIII	<10	<10	-	80	-	40	-	20	-	<10	-
XXXIV	<10	40	160	80	-	40	-	20	-	<10	-
XXXV	<10	<10	40	20	-	20	-	10	-	<10	-
XXXVI	<10	<10	40	40	-	20	-	<10	-	<10	-
XXXVII	<10	80	160	80	-	40	-	-	-	20	-
XXXVIII	<10	40	-	80		-	-	20	-	<10	-

[1] <10, detection limit; [2] - , no blood sample taken
