# Supplementary figures and images for: Vaccination against Borna Disease: Overview, Vaccine Virus Characterization and Investigation of Live and Inactivated Vaccines
Source: Viruses. 2022 Dec 2;14(12):2706. doi: 10.3390/v14122706 (PMC9788498; doi:10.3390/v14122706)

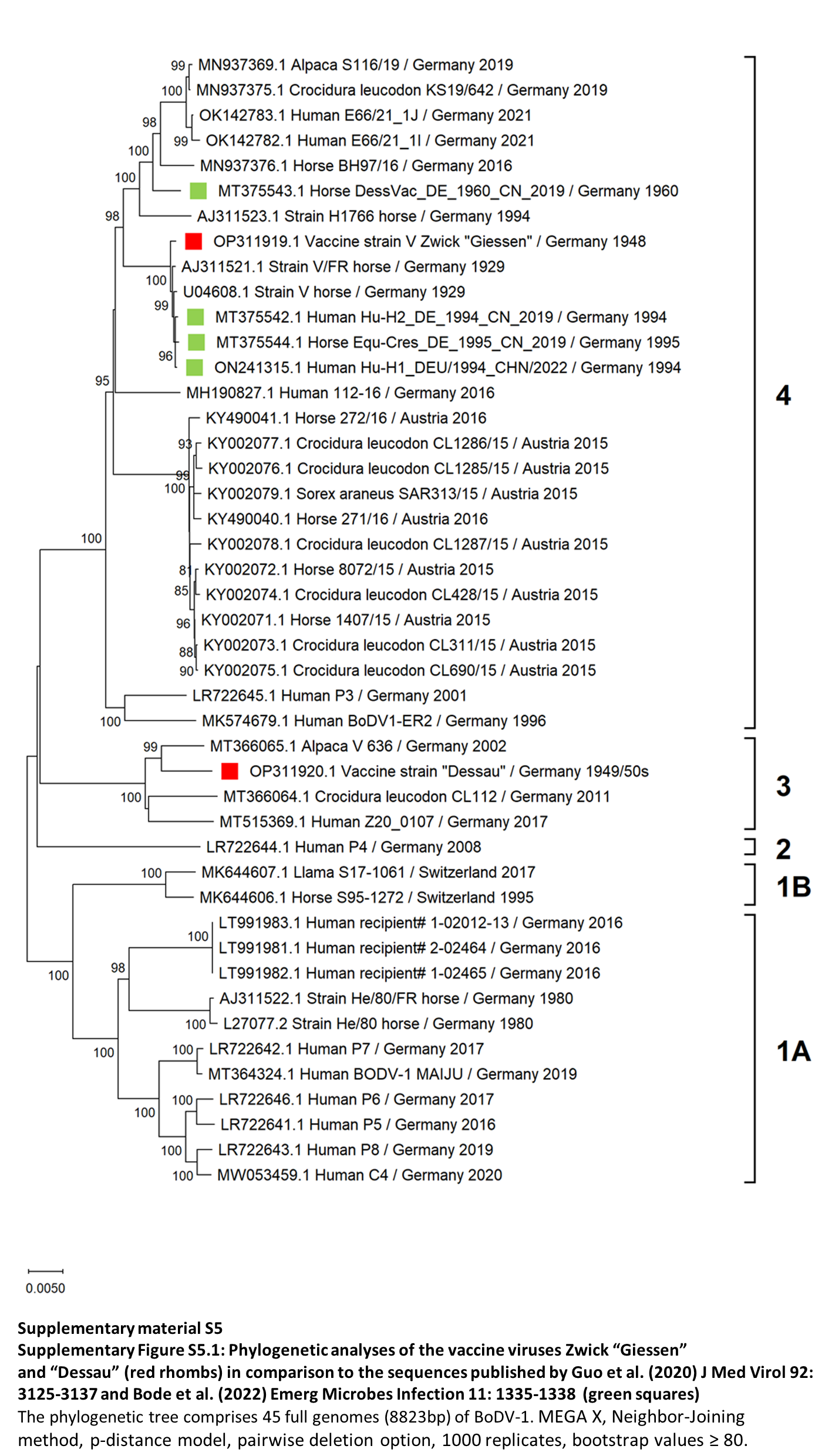

Supplement: Supplementary file 1 [file viruses-14-02706-s001.zip › Supplementary material S5.tif]
